# Immune regulation and blood–brain barrier permeability in cerebral small vessel disease: study protocol of the INflammation and Small Vessel Disease (INSVD) study – a multicentre prospective cohort study

**DOI:** 10.1136/bmjopen-2024-084303

**Published:** 2024-02-26

**Authors:** Audrey Low, Sanne van Winden, Lupei Cai, Roy P C Kessels, Marnix C Maas, Robin G Morris, Meritxell Nus, Daniel J Tozer, Anil Tuladhar, Anja van der Kolk, Rowan Wolters, Ziad Mallat, Niels P Riksen, Hugh Markus, Frank‐Erik de Leeuw

**Affiliations:** 1 Department of Clinical Neurosciences, University of Cambridge, Cambridge, UK; 2 Department of Neurology, Radboudumc, Nijmegen, The Netherlands; 3 Radboud University Donders Institute for Brain Cognition and Behaviour, Nijmegen, The Netherlands; 4 Vincent Van Gogh Instituut, Venray, The Netherlands; 5 Department of Radiology and Nuclear Medicine, Radboudumc, Nijmegen, The Netherlands; 6 Institute of Psychiatry, Psychology and Neuroscience (IoPPN), King's College London, London, UK; 7 Division of Cardiovascular Medicine, Department of Medicine, University of Cambridge, Cambridge, UK; 8 The Victor Phillip Dahdaleh Heart and Lung Research Institute, Section of Cardiorespiratory Medicine, Department of Medicine, University of Cambridge Medicine, Cambridge, UK; 9 Department of Internal Medicine, Radboudumc, Nijmegen, The Netherlands

**Keywords:** Aged, Stroke, IMMUNOLOGY, Magnetic resonance imaging, Old age psychiatry

## Abstract

**Introduction:**

The INflammation and Small Vessel Disease (INSVD) study aims to investigate whether peripheral inflammation, immune (dys)regulation and blood–brain barrier (BBB) permeability relate to disease progression in cerebral small vessel disease (SVD). This research aims to pinpoint specific components of the immune response in SVD relating to disease progression. This could identify biomarkers of SVD progression, as well as potential therapeutic targets to inform the development and repurposing of drugs to reduce or prevent SVD, cognitive decline and vascular dementia.

**Methods and analysis:**

INSVD is a prospective observational multicentre cohort study in individuals with symptomatic SVD. This longitudinal study combines comprehensive immunophenotyping of the peripheral blood immune compartment with advanced neuroimaging markers of SVD and BBB permeability. The main SVD marker of interest is white matter microstructure as determined by diffusion tensor imaging, a valuable marker of disease progression owing to its sensitivity to early alterations to white matter integrity. The research is being conducted in two sites—in the UK (Cambridge) and the Netherlands (Nijmegen)—with each site recruiting 100 participants (total n=200). Participants undergo clinical and cognitive assessments, blood draws, and brain MRI at baseline and 2-year follow-up.

**Ethics and dissemination:**

This study received ethical approval from the local ethics boards (UK: East of England—Cambridge Central Research Ethics Committee (REC) ref: 22/EE/00141, Integrated Research Application System (IRAS) ID: 312 747. Netherlands: Medical Research Ethics Committee (MREC) Oost-Nederland, ref: 2022-13623, NL-number: NL80258.091.22). Written informed consent was obtained from all subjects before the study. Any participant-derived benefits resulting from this research, such as new insights into disease mechanisms or possible novel therapies, will be disseminated to study participants, patient groups and members of the public.

**Trial registration number:**

NCT05746221.

**STRENGTHS AND LIMITATIONS OF THIS STUDY**

- Deep immunophenotyping of the peripheral blood immune compartment using state-of-the-art (epi)genetic, transcriptomic and proteomic techniques and functional assays.
- Longitudinal cohort study design to track disease progression.
- Advanced neuroimaging techniques to measure and map blood–brain barrier integrity across the brain.
- Multicentre study with comparable neuroimaging protocols on identical 3T MRI scanners in both sites.
- However, as deep immunophenotyping is only performed at baseline, this study is not able to assess whether changes in immune cell phenotype are predictive of disease progression.

## Introduction

Cerebral small vessel disease (SVD) is a common condition in older adults characterised by injury to the small penetrating vessels of the brain and accounts for a quarter of all strokes, both intracerebral haemorrhages and lacunar strokes.^1^ As well as resulting in vascular cognitive impairment (VCI),^2 3^ SVD is the major vascular pathology contributing to dementia.^4^ At present, there are no disease-modifying treatments for the resulting VCI, and treatments to reduce SVD are limited to controlling vascular risk factors such as hypertension.^5^ This lack of treatment options is mainly due to an incomplete understanding of the underlying pathophysiology of SVD, although emerging evidence highlights candidate mechanisms such as inflammation and blood-brain barrier (BBB) dysfunction.^6^

The presence of inflammation and immune dysregulation in SVD has been revealed both by blood markers of inflammation,^7^ and by ‘inflammation’ in the central nervous system, observed through neuroimaging (eg, activated microglia in the brain)^8 9^ and neuropathological examination.^10^ Recent exploratory studies showed an association between the cytokine production capacity of circulating monocytes and SVD progression.^11 12^ Innate immune cells can develop a persistent hyperinflammatory phenotype after brief stimulation by epigenetic reprogramming, which has been termed trained immunity.^13^ This also occurs in microglia and can contribute to neuropathology.^14^ We hypothesise that trained circulating and cerebral innate immune cells contribute to SVD progression. However, there remains much uncertainty about the underlying processes linking inflammation to SVD and, in particular, whether inflammation is causally related to disease progression.

Another closely related factor implicated in SVD pathogenesis is BBB permeability.^15–19^ Impaired BBB function is evidenced by albumin leakage into the cerebrospinal fluid or the imaging of gadolinium contrast agent leakage into the brain parenchyma.^15 19^ Normally, the BBB is selective of the blood components allowed to enter the central nervous system. In a dysfunctional state, however, BBB permeability is impaired, leading to the dysregulated leakage of substances into the brain, including harmful substances. Importantly, BBB dysfunction has been reported to be related to chronic unresolved inflammation and *in vivo* studies show a potential shared pathogenic pathway.^20^ As for inflammation, however, it is uncertain whether BBB permeability plays a causal role in SVD pathogenesis, and how it relates to both systemic and central inflammation in patients.

The INflammation and Small Vessel Disease (INSVD) study has been established to address these questions; in particular, to understand the relationship between inflammation and BBB permeability, and their potential relationship with SVD progression. INSVD is a longitudinal cohort study investigating whether systemic inflammation and immune (dys)function, and BBB permeability predict disease progression in SVD, with our primary SVD outcome measure being white matter microstructure as determined by diffusion tensor imaging (DTI), which is a valuable marker of disease progression owing to its sensitivity to early alterations to white matter integrity.^21^

Our overarching aim is to examine in detail the phenotype of circulating immune cells in patients with SVD to identify specific components of the dysregulated immune response which relate to disease progression. This could help identify biomarkers of disease progression and potential therapeutic targets for the development and repurposing of drugs to reduce SVD, VCI and vascular dementia. Study objectives are as follows:

### Primary objective

- To determine whether changes in immune cell function and phenotype identified in the systemic circulation predict disease progression in SVD, as measured by DTI-MRI.

### Secondary objectives

- To characterise the alteration in immune phenotype occurring in SVD and how these relate to clinical, neurocognitive and imaging parameters.
- To determine the relationships between systemic immune changes in SVD and BBB leakage.

We hypothesise that activation of the immune system plays a causal role in the progression of SVD, and that mediating processes resulting from this immune activation may involve BBB leakage and inflammation. This progressive damage then compromises structural and functional brain integrity, leading to progressive white matter damage and accelerated VCI.

## Methods and analysis

### Overview

**Figure 1:**
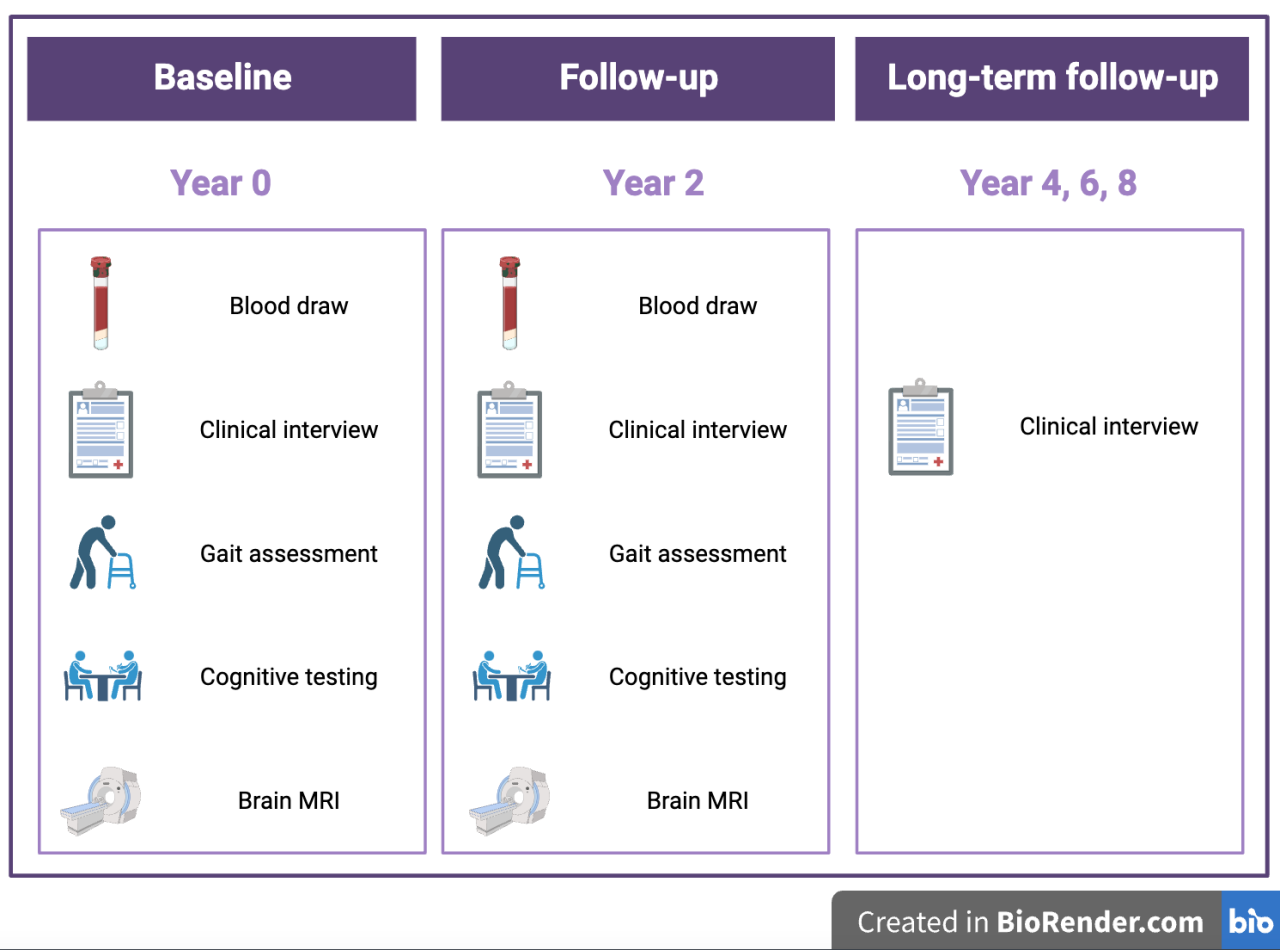
Overview of study components.

The INSVD study is a prospective multicentre observational cohort study in patients with SVD. The research will be conducted at two sites—Cambridge (UK) and Nijmegen (The Netherlands)—each recruiting 100 participants (total n=200). At baseline and 2-year follow-up, participants undergo clinical and cognitive assessments, blood draw and brain MRI. Follow-up will be continued for the next 6 years to track incident clinical events, among others (figure 1). Study recruitment began in August 2022 and study visits (baseline and 2-year follow-up) are projected to be completed by August 2026.

### Participants

#### Inclusion criteria

The study recruits individuals aged 40 and above with symptomatic SVD—this is defined as having a lacune on MRI according to the Standards for Reporting Vascular Changes on Neuroimaging (STRIVE) guidelines,^22 23^ in addition to at least one of the three following clinical presentations: (a) clinical lacunar stroke syndrome; (b) symptoms of cognitive impairment presumed to be due to SVD and (c) gait/motor impairment presumed to be due to SVD. Baseline study visits are performed after a minimum interval of 3 months after participants’ most recent stroke and at least 1 month after any systemic infection or vaccination.

#### Exclusion criteria

**Figure 2:**
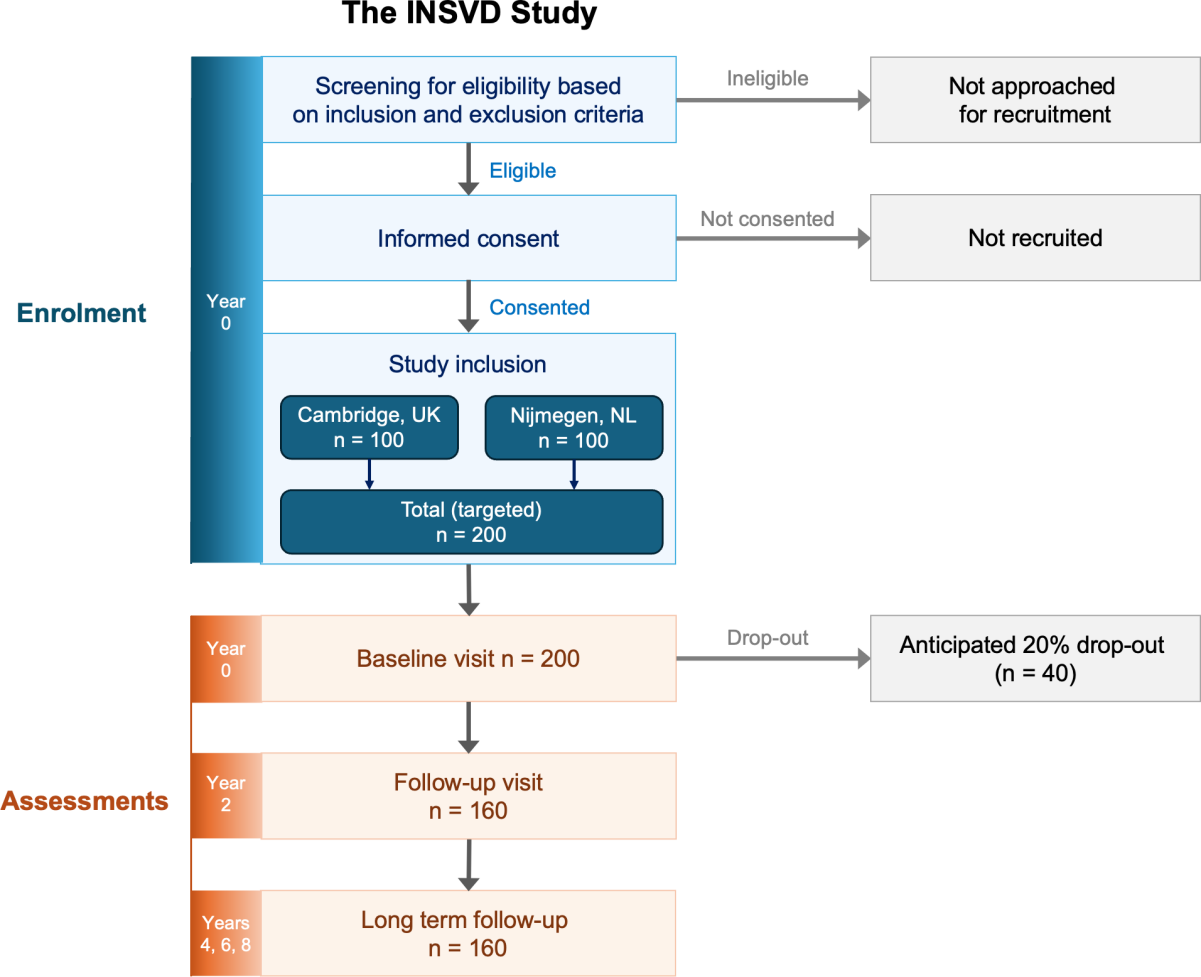
Study flow chart; based on planned sample sizes. INSVD, INflammation and Small Vessel Disease.

Potential participants are excluded if they have any MRI contraindication, or an estimated glomerular filtration rate (eGFR) <60 mL/min/1.73 m^2^ (UK) or <30 mL/min/1.73 m^2^ (Netherlands) within the past 3 months (figure 2). If eGFR has not been assessed within the last 3 months, this is measured as part of the research procedure. Other exclusion criteria include strokes caused by factors other than SVD (eg, cardioembolic source, carotid or vertebral artery stenosis >50%), another diagnosed chronic neurological condition (eg, Alzheimer’s disease, Parkinson’s disease), myocardial infarction in the past year, vasculitis, autoimmune or autoinflammatory disease, treatment with immunomodulating drugs, known monogenic causes of SVD, or limited life expectancy due to another illness or chronic condition which would make the 2-year follow-up difficult (eg, widespread malignancy).

#### Participant recruitment

At the Cambridge site, participants are screened and recruited from the clinical stroke service at Cambridge University Hospitals National Health Service Foundation Trust and patient registers of patients with SVD enrolled in previous research studies. Screening is conducted through electronic hospital records, discussions with the patient about relevant medical history, and through the local MRI safety questionnaire. ECG and carotid artery duplex scans have been performed as part of ischaemic stroke workup and are used to rule out stroke causes other than SVD.

At the Nijmegen site, participants are recruited from the Radboud University Nijmegen Diffusion tensor and Magnetic resonance imaging Cohort (RUN DMC) study,^24^ and the neurology outpatient clinic at Radboud University Medical Centre. Screening involves reviewing electronic hospital records and discussing medical history with the patient. ECG and carotid artery duplex scans are included as part of screening procedures, in case information on atrial fibrillation and large artery disease are not up to date or readily available from medical records.

#### Sample size calculation

Power calculations were based on previous findings demonstrating associations between circulating IL-6 and SVD progression,^25^ and our pilot data showing a correlation between IL-6 production in peripheral blood mononuclear cells (PBMC; after ex vivo toll-like receptor-2 (TLR2) stimulation) and SVD progression (white matter hyperintensity (WMH) progression) of r=0.22.^11^ To detect a correlation of r=0.22 at α=0.05 with 80% power (two-tailed test with Fisher formula), 160 participants with evaluable datasets are needed. To account for dropouts and unusable data (eg, failed MRI quality check) estimated at 20%, we are recruiting 200 participants.

### Measures

#### Clinical assessment

Demographics, medical history, medication use, cardiovascular risk factors (eg, diabetes, hypercholesterolaemia, hypertension) and behavioural risk factors (eg, smoking, alcohol consumption) are recorded based on electronic medical records and by interviewing participants at baseline. Clinical details of the most recent stroke are assessed, where relevant. Blood pressure and heart rate are measured after the participant has been seated comfortably for at least 5 min. Three measurements are collected, and means are calculated as the average of the last two readings. Height and weight are recorded to determine body mass index. Global disability is assessed using the Modified Rankin Score (mRS).^26^ At long-term follow-up visits, we record incident clinical events and changes in medication use and cardiovascular risk factors.

#### Neuroimaging

All participants undergo brain MRI, acquired at both sites on identical 3T scanners (MAGNETOM PrismaFit, Siemens Healthineers, Erlangen, Germany) with a 32-channel head coil. The acquisition protocol consists of the following sequences: three-dimensional (3D) T_1_-weighted magnetisation prepared rapid gradient echo (MPRAGE), 3D T_2_-weighted fluid-attenuated inversion recovery (FLAIR), 3D T_2_*-weighted susceptibility weighted imaging (SWI, Cambridge) or quantitative susceptibility mapping (QSM, Nijmegen), intravoxel incoherent motion imaging (IVIM, Nijmegen only), multishell diffusion-weighted imaging (DWI)^27–29^ and dynamic contrast enhanced (DCE) imaging (online supplemental table 1). DWI is acquired twice with opposing phase-encoding directions to enable correction for magnetic field and eddy current-induced geometric distortions. A B_0_ field map and an additional b=0 s/mm^2^ image with opposed phase-encoding polarity are acquired to correct for geometric distortions in IVIM imaging.

10.1136/bmjopen-2024-084303.supp1 Supplementary data

The study employs an optimised DCE-MRI protocol,^17^ which has been implemented in other studies conducted by the Cambridge research group.^9^ This uses a fast T_1_ mapping approach based on variable flip angle (VFA) spoiled gradient echo (SPGR) imaging, before and after the injection of a gadolinium-based contrast agent. Following one precontrast T_1_ mapping acquisition, a 0.025 mmol/kg dose of the gadolinium agent gadoterate meglumine (Dotarem, Guerbet, Villepinte, France) is administered by a medically qualified staff member. Postcontrast T_1_ mapping is started 1 min after administration and continued for approximately 20 min (seven time points) to map the signal changes that are used to calculate permeability.

T_1_-weighted MPRAGE images will be processed using standard imaging processing pipelines (eg, SIENAX) to obtain maps of grey matter, white matter and cerebrospinal fluid. Image output will be visually inspected for misclassifications, and then used in conjunction with WMH masks to estimate brain and tissue volumes. Diffusion imaging data are preprocessed using the FMRIB Software Library^30^ and MRtrix3 (mrtrix.org). Specifically, we will perform denoising, Gibbs artefact removal and correction for head motion, eddy current distortions and susceptibility-induced distortions. Diffusion data will be analysed using voxel-by-voxel maps of standard diffusion parameters (eg, MD, FA) to produce whole-tissue histograms. Additionally, peak width of skeletonised mean diffusivity (PSMD)^31^ will be derived from DTI. PSMD is calculated from tissue on a white matter skeleton and is a measure of the width of the MD histogram. For DCE analysis, T_1_ maps are first computed based on the VFA SPGR images using the scanner software (MAPIT package, Siemens Healthineers), correcting for B_1_ inhomogeneity using a separately acquired precontrast B_1_ map. The T_1_ maps are spatially coregistered for motion correction, and pharmacokinetic analysis is performed on a voxel-by-voxel basis using the Patlak model,^32^ yielding maps of K_trans_, the blood-to-tissue transfer constant. The T_1_ maps—and hence the K_trans_ maps—are also registered to the conventional images and used for analysis alongside manually delineated lesion masks. IVIM images will be processed and analysed as described previously.^33^

Participants will also be assessed on four SVD markers, namely WMH, lacunes, cerebral microbleeds (CMB) and perivascular spaces (PVS), according to the STRIVE-2 criteria.^23^ All scans will be visually assessed by trained raters, blinded to all demographic and clinical information. WMH maps will be obtained from FLAIR images using an automated tool. Lesion maps will be reviewed by a single trained rater blinded to all clinical information and manually corrected for misclassifications. Haemorrhages and isolated lacunes will be excluded from the WMH mask. WMH volumes will be extracted from the finalised lesion masks and normalised by total intracranial volume. WMH will also be assessed semiquantitatively through visual rating.^34^ Periventricular and deep WMH will be rated separately. Lacunes and PVS will be identified manually using T_1_-weighted and FLAIR images and delineated to produce lesion maps. PVS, CMB and lacunes will also be rated semiquantitatively using validated scales where available.^35^ PVS will be assessed separately in the basal ganglia and centrum semiovale to account for different underlying pathologies. CMB will be manually delineated using SWI (Cambridge) and QSM images (Nijmegen) to derive CMB lesion maps.

#### Blood samples

Non-fasting, venous blood samples are drawn at baseline and 2-year follow-up by qualified clinical personnel. Blood withdrawal is performed before MRI to ensure that the injected gadolinium-based MRI contrast agent does not interfere with laboratory tests.^36^ Blood samples are collected at approximately the same time of day for all participants to minimise circadian-related variation in inflammatory and coagulation markers.^37^ At baseline, blood is collected in EDTA tubes for plasma, serum tubes for serum and BD Vacutainer CPT tubes (BD Biosciences) for whole blood and PBMCs. Blood tubes are stored at room temperature and processed within 2 hours after collection for the following procedures:

1. PBMCs are isolated using sodium heparin CPT tubes following manufacturer recommendations and freshly stimulated for 24 hours with the TLR-4 agonist lipopolysaccharide (LPS), with the TLR-2 agonist Pam3Cys-Ser-(Lys)4, with LPS plus the inflammasome activator Nigericin, or with RPMI buffer as control. Supernatants are stored at −80°C for future analysis of cytokines and chemokines.
2. Comprehensive immunophenotyping of fresh whole blood is performed by using Cytometry by Time-Of-Flight (CyTOF).
3. Isolated PBMCs are viably frozen to allow future single-cell RNA sequencing (scRNA-seq), Assay of Transposase Accessible Chromatin with high-throughput sequencing (ATAC-seq) and regulatory T cell (Treg) suppression assays. ScRNA-seq, ATAC-seq and Treg suppression assays will be performed after completion of the study in subgroups of patients with the highest and lowest progression rates of SVD.
4. A panel of circulating inflammatory markers is measured in serum using the Olink proteomics platform (Olink Proteomics AB, Uppsala, Sweden).

At 2-year follow-up, blood will be collected in EDTA tubes and processed according to manufacturer’s instructions. Plasma will be stored at −80°C for future analysis of circulating inflammatory and coagulation markers.

#### Neuropsychological assessment

At baseline and 2-year follow-up, participants undergo cognitive assessment using a test battery designed to capture characteristic cognitive impairment in SVD (online supplemental table 2). Tests have been selected that are validated and normed for use in both countries and languages (English, Dutch); for a more extensive description of each test see Lezak *et al*.^38^ Premorbid intelligence is assessed using the UK-developed National Adult Reading Test (NART-Revised) in Cambridge, and its Dutch equivalent (Dutch Adult Reading Test) in Nijmegen. Processing speed is measured using the Symbol Digit Modalities Test (Written) and the Trail Making Test A. Working memory is measured using the forward and backward conditions of the Digit Span subtest of the Wechsler Adult Intelligence Scale-Fourth Edition. Executive function is assessed using the Trail Making Test B and the Brixton Spatial Anticipation Test. Visuospatial ability is evaluated using the Rey-Osterrieth Complex Figure Test (RCFT)-Copy trial. Memory is measured using the RCFT-Recall trial and the Story Recall subtest from the Rivermead Behavioural Memory Test-3. The results of all tests will be converted to age-adjusted, education-adjusted and/or sex-adjusted standard scores using published country-specific normative data.

Self-administered questionnaires to assess psychological symptoms and social functioning are performed at the beginning of study visits to avoid responses being impacted by tiredness and affective changes from the clinical interview and cognitive tests. Depressive symptoms are assessed using the 30-item Geriatric Depression Scale.^39^ Apathy is evaluated using the Apathy Evaluation Scale.^40^ Fatigue is assessed using the Fatigue Severity Scale.^41^ Social cognition is assessed using the Social Norms Questionnaire.^42^

#### Gait assessment

Gait assessment is carried out at baseline and 2-year follow-up using the Timed Up and Go (TUG) test and the 6 m walk test. The TUG assesses gait performance and balance and is measured as the time (seconds) it takes a participant to rise from a chair, walk 3 m, turn around, walk back to the chair and sit back down. The 6 m walk test determines gait speed (metres/seconds). Participants have to walk a distance of 10 m, and gait speed is assessed over a 6 m distance by timing the participant from the 2 m point to the 8 m point. Participants are instructed to walk at a comfortable pace during both tests. The use of walking aids is recorded, if applicable. Both tests include a practice trial, followed by three timed trials which are averaged.

### Data analysis

Peak height of the MD histogram was selected as the primary endpoint, given our past demonstration that it is the most sensitive DTI marker for SVD progression.^43^ Based on longitudinal MRI data from the SCANS study, the yearly rate of change of MD peak height was −3.72×10^−4^ mm^2^/s (SD 3.12×10^−5^), which corresponds to an annual percentage change of −2.44%.^43^ This would equate to a change of −4.88% over the 2-year follow-up period of the INSVD study.

Our primary analysis will test whether baseline measures of inflammatory and immune markers predict SVD progression as measured by MD peak height. We assume a linear relationship between baseline levels of coagulation and inflammatory markers and the change in MD peak height. Therefore, we will run linear regression models and adjust for age, sex, education and other conventional MRI markers of SVD, where appropriate.

As our secondary objectives, we will assess (1) the relationship between inflammatory and immune markers and BBB permeability, both globally (cortical and subcortical volumes) and regionally (hotspot volumes) and (2) whether baseline levels of inflammatory and immune markers are associated with other imaging parameters, as well as cognitive and clinical outcomes. Similar to our primary analyses, we will conduct linear regression modelling to test these associations.

### Patient and public involvement

This research has been informed through consultation with external stakeholders, including patients and their families, both in the UK and Netherlands. In joint initiatives with the Stroke Association (UK), SVD and cognitive decline have been identified as major priorities for patients with stroke. In a joint funder working group attended by both patients and their families, funders and health professionals, research into the mechanisms underlying SVD was identified as a top priority. Study protocols were developed in partnership with local stroke support groups who advised on the study and logistics of implementation. To ensure that participants understand the study clearly before enrolment, the participant information sheet was reviewed by two patient volunteers, whose comments were incorporated into the final document.

### Ethics and dissemination

The study is conducted in accordance with the principles of the Declaration of Helsinki, the conditions and principles of Good Clinical Practice, and the applicable local regulatory requirements and laws. The project is funded by a joint grant from the British Heart Foundation and Dutch Heart Foundation (ref: SP/F/22/150028) and jointly sponsored by the Cambridge University Hospitals Foundation NHS Trust and the University of Cambridge in the UK, and the Radboud University Medical Centre in the Netherlands. Any participant-derived benefits resulting from this research, such as new insights into disease mechanisms or possible novel therapies, will be disseminated to study participants at patient groups and to members of the public through standardised communication platforms, including research journals and disease-specific national patient websites.

This study protocol has been registered on ClinicalTrials.gov (NCT05746221) and received ethical approval from the local ethics board (UK: East of England—Cambridge Central Research Ethics Committee (REC) ref: 22/EE/00141, Integrated Research Application System (IRAS) ID: 312 747. NL: Medical Research Ethics Committee (MREC) Oost-Nederland, file number: 2022-13623, NL-number: NL80258.091.22).

## Supplementary Material

Reviewer comments

Author's manuscript
