# Supplementary material for: Immune regulation and blood–brain barrier permeability in cerebral small vessel disease: study protocol of the INflammation and Small Vessel Disease (INSVD) study – a multicentre prospective cohort study
Source: BMJ Open. 2024 Feb 26;14(2):e084303. doi: 10.1136/bmjopen-2024-084303 (PMC10900331; doi:10.1136/bmjopen-2024-084303)
Supplement: Supplementary data [file bmjopen-2024-084303supp001.pdf]

Supplementary Table 1. MRI acquisition parameters.

	T1		FLAIR		T2* gradient echo		DTI		IVIM		DCE	
					SWI	QSM						
**Study site**	C	N	C	N	C	N	C	N	C	N	C	N
**Sequence**	3D MPRAGE		3D SPACE		3D SPGR		Single-shot spin-echo EPI		NA	Single-shot spin-echo EPI	3D SPGR	
**TR,** ms	2300		6000	5000	29	40	2433		NA	3386	15	
**TE,** ms	2.98		383	394	20	3.5, 8.6, 13.7, 18.9, 24.0, 29.2, 34.3	75.6		NA	60.6	1.75	
**TI,** ms	900		2100	1800	NA	NA	NA		NA	NA	NA	
**Flip angle**	9°		multiple	multiple	15°	15°	80°		NA	90°	5°, 10°, 15°, 21°, 26°	
**Fat suppression**	None		None	None	None	WEX	FatSat		NA	FatSat	None	
**FOV** mm²	256×248		256×256	250×250	256×256	256×256	192×192		NA	212×212	240×240	
**Voxel size** mm³	1×1×1		1×1×1	0.5×0.5×1	0.5×0.5×2	1×1×1	1.7×1.7×1.8		NA	2×2×2	1.9×1.9×4.0	
**Slices**	176		192	176	72	176	76		NA	74	16	
**Slice orientation**	SAG		SAG	SAG	TRA	SAG	TRA		NA	TRA	TRA	
**Diffusion b-values** s/mm²	NA		NA	NA	NA	NA	0, 300, 1000, 2000		NA	0, 5, 10, 15, 20, 30, 40, 50, 60, 100, 200, 400, 600, 800, 1000	NA	
**Diffusion gradient directions**	NA		NA	NA	NA	NA	6, 8, 30, 60		NA	6	NA	
**Parallel imaging accel. Factor**	2	3	2	2	2	6	NA		NA	2	NA	
**Multiband accel. Factor**	NA		NA	NA	NA	NA	4		NA	2	NA	
**Acquisition time,** mm:ss	05:12	03:01	05:44	04:42	04:35	05:06	04:28		NA	05:39	21:12	
**Temporal res.** mm:ss	NA		NA	NA	NA	NA	NA		NA	NA	02:39	

Abbreviations: C = Cambridge, DCE = dynamic contrast-enhanced MRI, DTI = diffusion tensor imaging (multi-shell with 98 gradient directions and 6 b=0 values), *FLAIR = fluid-attenuated inversion recovery, FOV = field of view, GRAPPA = GeneRalized Autocalibrating Partial Parallel Acquisition, IVIM = Intravoxel Incoherent Motion, N = Nijmegen, SAG = sagittal, SWI = susceptibility-weighted imaging, TI = inversion time, TE = echo time, TR = repetition time, TRA = transversal*.

Supplementary Table 2. Neuropsychological assessment measures.

Measured construct	Instrument	Mode of administration	Type of measure
Premorbid intelligence	National/Dutch Adult Reading Test	Verbal	# correctly pronounced
Processing speed	Trail Making Test A	Written	time, # errors
	Symbol Digit Modalities Test	Written	# completed in 90s
Working memory	Digit Span Forwards and Backwards	Verbal	# correct trials
	Story Recall – Immediate Recall	Verbal	# correct elements recalled
Executive function	Trail Making Test B	Verbal	time, # errors
	Brixton Spatial Anticipation Test	Verbal	# errors
Visuospatial ability	Rey-Osterrieth Complex Figure Copy	Written	# correct elements drawn
Memory	Story Recall – Delayed Recall	Verbal	# correct elements recalled
	Rey-Osterrieth Complex Figure Recall	Written	# correct elements recalled
Depression	Geriatric Depression Scale (30-item version)	Self-administered questionnaire	Yes/No
Apathy	Apathy Evaluation Scale – Self	Self-administered questionnaire	4-point scale
	Apathy Evaluation Scale – Informant	Informant-rated questionnaire	4-point scale
Fatigue	Fatigue Severity Scale	Self-administered questionnaire	7-point scale
Social cognition	Social Norms Questionnaire	Self-administered questionnaire	Yes/No

Low A, *et al. BMJ Open* 2024; 14:e084303. doi: 10.1136/bmjopen-2024-084303
