# Supplementary material for: Immune regulation and blood–brain barrier permeability in cerebral small vessel disease: study protocol of the INflammation and Small Vessel Disease (INSVD) study – a multicentre prospective cohort study
Source: BMJ Open. 2024 Feb 26;14(2):e084303. doi: 10.1136/bmjopen-2024-084303 (PMC10900331; doi:10.1136/bmjopen-2024-084303)
Supplement: Author's manuscript [file bmjopen-2024-084303.draft_revisions.pdf]

BMJ Open

BMJ Open is committed to open peer review. As part of this commitment we make the peer review history of every article we publish publicly available.

When an article is published we post the peer reviewers' comments and the authors' responses online. We also post the versions of the paper that were used during peer review. These are the versions that the peer review comments apply to.

The versions of the paper that follow are the versions that were submitted during the peer review process. They are not the versions of record or the final published versions. They should not be cited or distributed as the published version of this manuscript.

BMJ Open is an open access journal and the full, final, typeset and author-corrected version of record of the manuscript is available on our site with no access controls, subscription charges or pay-per-view fees (http://bmjopen.bmj.com).

If you have any questions on BMJ Open's open peer review process please email info.bmjopen@bmj.com

Immune regulation and blood-brain barrier permeability in cerebral small vessel disease – protocol of the INflammation and Small Vessel Disease (INSVD) study, a multicentre prospective cohort study

Journal:	*BMJ Open*
Manuscript ID	bmjopen-2024-084303
Article Type:	Protocol
Date Submitted by the Author:	15-Jan-2024
Complete List of Authors:	Low, Audrey; University of Cambridge, Department of Clinical Neurosciences<br>van Winden, Sanne; Radboudumc, Department of Neurology; Radboud University Donders Institute for Brain Cognition and Behaviour<br>Cai, Lupei; University of Cambridge, Department of Clinical Neurosciences<br>Kessels, Roy; Radboud University Donders Institute for Brain Cognition and Behaviour; Vincent Van Gogh Instituut<br>Maas, Marnix C.; Radboudumc, Department of Radiology and Nuclear Medicine<br>Morris, Robin; King's College London, Institute of Psychiatry, Psychology and Neuroscience (IoPPN)<br>Nus, Meritxell; University of Cambridge, Division of Cardiovascular Medicine, Department of Medicine<br>Tozer, Daniel; University of Cambridge, Department of Clinical Neurosciences<br>Tuladhar, Anil; Radboudumc, Department of Neurology; Radboud University Donders Institute for Brain Cognition and Behaviour<br>van der Kolk, Anja; Radboudumc, Department of Radiology and Nuclear Medicine<br>Wolters, Rowan; Radboudumc, Department of Neurology<br>Mallat, Ziad; University of Cambridge Medicine, The Victor Phillip Dahdaleh Heart and Lung Research Institute, Section of Cardiorespiratory Medicine, Department of Medicine<br>Riksen, Niels; Radboudumc, Department of Internal Medicine<br>Markus, Hugh; University of Cambridge, Department of Clinical Neurosciences<br>de Leeuw, Frank-Erik ; Radboudumc, Department of Neurology
Keywords:	Aged, Stroke < NEUROLOGY, IMMUNOLOGY, Magnetic resonance imaging < RADIOLOGY & IMAGING, Old age psychiatry < PSYCHIATRY

Immune regulation and blood-brain barrier permeability in cerebral small vessel disease – protocol of the INflammation and Small Vessel Disease (INSVD) study, a multicentre prospective cohort study

Audrey Low[1,*], Sanne van Winden[2,3,*], Lupei Cai[1], Roy P.C. Kessels[3,4,5], Marnix C. Maas[6], Robin Morris[7], Meritxell Nus[8], Daniel J. Tozer[1], Anil M. Tuladhar[2,3], Anja van der Kolk[6], Rowan Wolters[2], Ziad Mallat[8], Niels P. Riksen[9], Hugh S. Markus[1,†], Frank-Erik de Leeuw[2,3,†]

Affiliations

[1] Stroke Research Group, Department of Clinical Neurosciences, University of Cambridge, UK

[2] Department of Neurology, Radboud Research Institute for Medical Innovation, Radboud University Medical Centre, Nijmegen, The Netherlands

[3] Donders Institute for Brain, Cognition, and Behaviour, Radboud University, Nijmegen, The Netherlands

[4] Department of Medical Psychology and Radboudumc Alzheimer Centre, Radboud University Medical Centre, Nijmegen, The Netherlands

[5] Vincent van Gogh Institute for Psychiatry, Venray, The Netherlands

[6] Department of Medical Imaging, Radboud University Medical Centre, Nijmegen, The Netherlands

[7] Department of Psychology, King's College London, Institute of Psychiatry, Psychology and Neuroscience, UK

[8] The Victor Phillip Dahdaleh Heart and Lung Research Institute, Section of Cardiorespiratory Medicine, Department of Medicine, University of Cambridge, UK

[9] Department of Internal Medicine, Radboud University Medical Centre, Nijmegen, The Netherlands

* joint first authors

† joint senior authors

Corresponding Author

Audrey Low

Department of Clinical Neurosciences, Neurology Unit, University of Cambridge

School of Clinical Medicine, R3, Box 83, Cambridge Biomedical Campus

Cambridge, Cambridgeshire, United Kingdom, CB2 0SP

Email: al927@cam.ac.uk

MeSH keywords: Aged, Stroke, Immunology, Magnetic Resonance Imaging, Old age psychiatry

Word count: 3182

For peer review only

ABSTRACT

Introduction

The INflammation and Small Vessel Disease (INSVD) study aims to investigate whether peripheral inflammation, immune (dys)regulation and blood-brain barrier (BBB) permeability relate to disease progression in cerebral small vessel disease (SVD). This research aims to pinpoint specific components of the immune response in SVD relating to disease progression. This could identify biomarkers of SVD progression, as well as potential therapeutic targets to inform the development and repurposing of drugs to reduce or prevent SVD, cognitive decline, and vascular dementia.

Methods and analysis

INSVD is a prospective observational multicentre cohort study in individuals with symptomatic SVD. This longitudinal study combines comprehensive immunophenotyping of the peripheral blood immune compartment with advanced neuroimaging markers of SVD and BBB permeability. The main SVD marker of interest is white matter microstructure as determined by diffusion tensor imaging (DTI), a valuable marker of disease progression owing to its sensitivity to early alterations to white matter integrity. The research is being conducted in two sites – in the United Kingdom (Cambridge) and the Netherlands (Nijmegen) – with each site recruiting 100 participants (total n=200). Participants undergo clinical and cognitive assessments, blood draws, and brain MRI at baseline and two-year follow-up.

Ethics and dissemination

This study received ethical approval from the local ethics board (UK: East of England - Cambridge Central Research Ethics Committee (REC) ref: 22/EE/00141, Integrated Research Application System (IRAS) ID: 312747. NL: Medical Research Ethics Committee (MREC) Oost-Nederland, ref: 2022-13623, NL-number: NL80258.091.22). Written informed consent was obtained from all subjects before the study. Any participant-derived benefits resulting from this research, such as new insights into disease mechanisms or possible novel therapies, will be disseminated to study participants, patient groups, and members of the public.

Fundings and public registration

The INSVD study is funded by a joint grant from the British Heart Foundation and Dutch Heart Foundation (SP/F/22/150028) and has been registered on ClinicalTrials.gov (NCT05746221).

For peer review only - http://bmjopen.bmj.com/site/about/guidelines.xhtml

ARTICLE SUMMARY

Strengths and limitations of this study

- Strength: Deep immunophenotyping of the peripheral blood immune compartment using state-of-the-art (epi)genetic, transcriptomic and proteomic techniques and functional assays
- Strength: Longitudinal cohort study design to track disease progression
- Strength: Advanced neuroimaging techniques to measure and map blood-brain barrier integrity across the brain
- Strength: Multicentre study with comparable neuroimaging protocols on identical 3T MRI scanners in both sites
- Limitation: Deep immunophenotyping only performed at baseline, thus this study is not able to assess whether *changes* in immune cell phenotype are predictive of disease progression

For peer review only

1. INTRODUCTION

Cerebral small vessel disease (SVD) is a common condition in older adults characterised by injury to the small penetrating vessels of the brain, and accounts for a quarter of all strokes, both intracerebral haemorrhages and lacunar strokes.[1] As well as resulting in vascular cognitive impairment (VCI),[2,3] SVD is the major vascular pathology contributing to dementia.[4] At present, there are no disease-modifying treatments for the resulting VCI, and treatments to reduce SVD are limited to controlling vascular risk factors like hypertension.[5] This lack of treatment options is mainly due to an incomplete understanding of the underlying pathophysiology of SVD, although emerging evidence highlights candidate mechanisms such as inflammation and blood-brain barrier (BBB) dysfunction.[6]

The presence of inflammation and immune dysregulation in SVD has been revealed both by blood markers of inflammation,[7] and by 'inflammation' in the central nervous system, observed through neuroimaging (e.g., activated microglia in the brain)[8,9] and neuropathological examination.[10] Recent exploratory studies showed an association between the cytokine production capacity of circulating monocytes and SVD progression.[11,12] Innate immune cells can develop a persistent hyperinflammatory phenotype after brief stimulation by epigenetic reprogramming, which has been termed trained immunity.[13] This also occurs in microglia and can contribute to neuropathology.[14] We hypothesize that trained circulating and cerebral innate immune cells contribute to SVD progression. However, there remains much uncertainty about the underlying processes linking inflammation to SVD and, in particular, whether inflammation is causally related to disease progression.

Another closely related factor implicated in SVD pathogenesis is BBB permeability.[15–19] Impaired BBB function is evidenced by albumin leakage into the cerebrospinal fluid or the imaging of gadolinium contrast agent leakage into the brain parenchyma.[15,19] Normally, the BBB is selective of the blood components allowed to enter the central nervous system. In a dysfunctional state, however, BBB permeability is impaired, leading to the dysregulated leakage of substances into the brain, including harmful substances. Importantly, BBB dysfunction has been reported to be related to chronic unresolved inflammation and *in vivo* studies show a potential shared pathogenic pathway.[20] As for inflammation, however, it is uncertain whether BBB permeability plays a causal role in SVD pathogenesis, and how it relates to both systemic and central inflammation in patients.

The *INflammation and Small Vessel Disease* (INSVD) study has been established to address these questions; in particular, to understand the relationship between inflammation and BBB permeability, and

For peer review only - http://bmjopen.bmj.com/site/about/guidelines.xhtml

their potential relationship with SVD progression. INSVD is a longitudinal cohort study investigating whether systemic inflammation and immune (dys)function, and BBB permeability predict disease progression in SVD, with our primary SVD outcome measure being white matter microstructure as determined by diffusion tensor imaging (DTI), which is a valuable marker of disease progression owing to its sensitivity to early alterations to white matter integrity.[21]

Our overarching aim is to examine in detail the phenotype of circulating immune cells in patients with SVD to identify specific components of the dysregulated immune response which relate to disease progression. This could help identify biomarkers of disease progression and potential therapeutic targets for the development and repurposing of drugs to reduce SVD, VCI, and vascular dementia. Study objectives are as follows:

Primary objective

- To determine whether changes in immune cell function and phenotype identified in the systemic circulation predict disease progression in SVD, as measured by DTI-MRI

Secondary objectives

- To characterise the alteration in immune phenotype occurring in SVD and how these relate to clinical, neurocognitive and imaging parameters
- To determine the relationships between systemic immune changes in SVD and BBB leakage

We hypothesise that activation of the immune system plays a causal role in the progression of SVD, and that mediating processes resulting from this immune activation may involve BBB leakage and inflammation. This progressive damage then compromises structural and functional brain integrity, leading to progressive white matter damage and accelerated VCI.

For peer review only - http://bmjopen.bmj.com/site/about/guidelines.xhtml

2. METHODS AND ANALYSIS

2.1. Overview

The INSVD study is a prospective multicentre observational cohort study in patients with SVD. The research will be conducted at two sites – Cambridge (UK), and Nijmegen (The Netherlands) – each recruiting 100 participants (total n=200). At baseline and two-year follow-up, participants undergo clinical and cognitive assessments, blood draw, and brain MRI. Follow-up will be continued for the next six years to track incident clinical events, among others (**Figure 1**).

[Figure 1 here]

2.2. Participants

2.2.1. Inclusion criteria

The study recruits individuals aged 40 and above with symptomatic SVD – this is defined as having a lacune on MRI according to the Standards for Reporting Vascular Changes on Neuroimaging (STRIVE) guidelines,[22,23] in addition to at least one of the three following clinical presentations: (a) clinical lacunar stroke syndrome; (b) symptoms of cognitive impairment presumed to be due to SVD; (c) gait/motor impairment presumed to be due to SVD. Baseline study visits are performed after a minimum interval of three months after participants' most recent stroke and at least one month after any systemic infection or vaccination.

2.2.2. Exclusion criteria

Potential participants are excluded if they have any MRI contraindication, or an estimated glomerular filtration rate (eGFR) <60ml/min/1.73m^2 (UK) or <30ml/min/1.73m^2 (Netherlands) within the past three months. If eGFR has not been assessed within the last three months, this is measured as part of the research procedure. Other exclusion criteria include strokes caused by factors other than SVD (e.g., cardioembolic source, carotid or vertebral artery stenosis >50%), another diagnosed chronic neurological condition (e.g., Alzheimer's disease, Parkinson's disease), myocardial infarction in the past year, vasculitis, autoimmune or autoinflammatory disease, treatment with immunomodulating drugs, known monogenic causes of SVD, or limited life expectancy due to another illness or chronic condition which would make the two-year follow-up difficult (e.g., widespread malignancy).

For peer review only - http://bmjopen.bmj.com/site/about/guidelines.xhtml

2.2.3. Participant recruitment

At the Cambridge site, participants are screened and recruited from the clinical stroke service at Cambridge University Hospitals NHS Foundation Trust and patient registers of SVD patients enrolled in previous research studies (Figure 2). Screening is conducted through electronic hospital records, discussions with the patient about relevant medical history, and through the local MRI safety questionnaire. ECG and carotid artery duplex scans have been performed as part of ischemic stroke workup, and are used to rule out stroke causes other than SVD.

At the Nijmegen site, participants are recruited from the RUN DMC study cohort,[24] and the neurology outpatient clinic at Radboud University Medical Centre. Screening involves reviewing electronic hospital records and discussing medical history with the patient. ECG and carotid artery duplex scans are included as part of screening procedures, in case information on atrial fibrillation and large artery disease are not up to date or readily available from medical records.

[Figure 2 here]

2.2.4. Sample size calculation

Power calculations were based on previous findings demonstrating associations between circulating IL-6 and SVD progression,[25] and our pilot data showing a correlation between IL-6 production in peripheral blood mononuclear cells (PBMC; after ex vivo toll-like receptor-2 (TLR2) stimulation) and SVD progression (white matter hyperintensity (WMH) progression) of $r=0.22$.[11] To detect a correlation of $r=0.22$ at $\alpha=0.05$ with 80% power (two-tailed test with Fisher formula), 160 participants with evaluable datasets are needed. To account for dropouts and unusable data (e.g., failed MRI quality check) estimated at 20%, we are recruiting 200 participants.

For peer review only - http://bmjopen.bmj.com/site/about/guidelines.xhtml

2.3. Measures

2.3.1. Clinical assessment

Demographics, medical history, medication use, cardiovascular risk factors (e.g., diabetes, hypercholesterolaemia, hypertension), and behavioural risk factors (e.g., smoking, alcohol consumption) are recorded based on electronic medical records and by interviewing participants at baseline. Clinical details of the most recent stroke are assessed, where relevant. Blood pressure and heart rate are measured after the participant has been seated comfortably for at least five minutes. Three measurements are collected, and means are calculated as the average of the last two readings. Height and weight are recorded to determine body mass index. Global disability is assessed using the Modified Rankin Score (mRS).[26] At long-term follow-up visits, we record incident clinical events and changes in medication use and cardiovascular risk factors.

2.3.2. Neuroimaging

All participants undergo brain MRI, acquired at both sites on identical 3T scanners (MAGNETOM PrismaFit, Siemens Healthineers, Erlangen, Germany) with a 32-channel head coil. The acquisition protocol consists of the following sequences: three-dimensional (3D) T_1-weighted magnetization prepared rapid gradient echo (MPRAGE), 3D T_2-weighted fluid-attenuated inversion recovery (FLAIR), 3D T_2*-weighted susceptibility weighted imaging (SWI, Cambridge) or quantitative susceptibility mapping (QSM, Nijmegen), intravoxel incoherent motion imaging (IVIM, Nijmegen only), multi-shell diffusion-weighted imaging (DWI),[34–36] and dynamic contrast enhanced (DCE) imaging (**Supplementary Table 1**). DWI is acquired twice with opposing phase-encoding directions to enable correction for magnetic field and eddy current-induced geometric distortions. A B_0 field map and an additional b=0 s/mm^2 image with opposed phase-encoding polarity are acquired to correct for geometric distortions in IVIM imaging.

The study employs an optimised DCE-MRI protocol[17] which has been implemented in other studies conducted by the Cambridge research group.[9] This uses a fast T_1 mapping approach based on variable flip angle (VFA) spoiled gradient echo (SPGR) imaging, before and after the injection of a gadolinium-based contrast agent. Following one pre-contrast T_1 mapping acquisition, a 0.025mmol/kg dose of the gadolinium agent gadoterate meglumine (Dotarem®, Guerbet, Villepinte, France) is administered by a medically qualified staff member. Post-contrast T_1 mapping is started one minute after

For peer review only - http://bmjopen.bmj.com/site/about/guidelines.xhtml

administration and continued for approximately 20 minutes (7 time points) to map the signal changes that are used to calculate permeability.

T_1-weighted MPRAGE images will be processed using standard imaging processing pipelines (e.g., SIENAX) to obtain maps of grey matter, white matter, and cerebrospinal fluid. Image output will be visually inspected for misclassifications, and then used in conjunction with WMH masks to estimate brain and tissue volumes. Diffusion imaging data are pre-processed using the FMRIB Software Library (FSL)[37] and MRtrix3 (mrtrix.org). Specifically, we will perform denoising, Gibbs artefact removal and correction for head motion, eddy current distortions and susceptibility-induced distortions. Diffusion data will be analysed using voxel-by-voxel maps of standard diffusion parameters (e.g., MD, FA) to produce whole-tissue histograms. Additionally, Peak Width of Skeletonized Mean Diffusivity (PSMD)[38] will be derived from DTI. PSMD is calculated from tissue on a white matter skeleton and is a measure of the width of the MD histogram. For DCE analysis, T_1 maps are first computed based on the VFA SPGR images using the scanner software (MAPIT package, Siemens Healthineers), correcting for B_1 inhomogeneity using a separately acquired pre-contrast B_1 map. The T1 maps are spatially coregistered for motion correction, and pharmacokinetic analysis is performed on a voxel-by-voxel basis using the Patlak model,[39] yielding maps of K_{trans}, the blood-to-tissue transfer constant. The T_1 maps – and hence the K_{trans} maps – are also registered to the conventional images and used for analysis alongside manually delineated lesion masks. IVIM images will be processed and analysed as described previously.[40]

Participants will also be assessed on four SVD markers, namely WMH, lacunes, CMB, and PVS, according to the STRIVE-2 criteria.[23] All scans will be visually assessed by trained raters, blinded to all demographic and clinical information. WMH maps will be obtained from FLAIR images using an automated tool. Lesion maps will be reviewed by a single trained rater blinded to all clinical information and manually corrected for misclassifications. Haemorrhages and isolated lacunes will be excluded from the WMH mask. WMH volumes will be extracted from the finalised lesion masks and normalised by total intracranial volume. WMH will also be assessed semi-quantitatively through visual rating.[41] Periventricular and deep WMH will be rated separately. Lacunes and PVS will be identified manually using T_1-weighted and FLAIR images and delineated to produce lesion maps. PVS, CMB and lacunes will also be rated semi-quantitatively using validated scales where available.[42] PVS will be assessed separately in the basal ganglia and centrum semiovale to account for different underlying pathologies. CMB will be manually delineated using SWI (Cambridge) and QSM images (Nijmegen) to derive CMB lesion maps.

For peer review only - http://bmjopen.bmj.com/site/about/guidelines.xhtml

2.3.3. Blood samples

Non-fasting, venous blood samples are drawn at baseline and two-year follow-up by qualified clinical personnel. Blood withdrawal is performed before MRI to ensure that the injected gadolinium-based MRI contrast agent does not interfere with laboratory tests.[32] Blood samples are collected at approximately the same time of day for all participants to minimise circadian-related variation in inflammatory and coagulation markers.[33] At baseline, blood is collected in EDTA tubes for plasma, serum tubes for serum, and BD Vacutainer CPT tubes (BD Biosciences) for whole blood and PBMCs. Blood tubes are stored at room temperature and processed within 2 hours after collection for the following procedures:

1. PBMCs are isolated using sodium heparin CPT tubes following manufacturer recommendations and freshly stimulated for 24 hours with the TLR-4 agonist lipopolysaccharide (LPS), with the TLR-2 agonist Pam3Cys-Ser-(Lys)4, with LPS plus the inflammasome activator Nigericin, or with RPMI buffer as control. Supernatants are stored at -80°C for future analysis of cytokines and chemokines.

2. Comprehensive immunophenotyping of fresh whole blood is performed using Cytometry by Time-Of-Flight (CyTOF).

3. Isolated PBMCs are viably frozen to allow future single-cell RNA sequencing (scRNA-seq), Assay of Transposase Accessible Chromatin with high-throughput sequencing (ATAC-seq) and regulatory T cell (Treg) suppression assays. ScRNA-seq, ATAC-seq and Treg suppression assays will be performed after completion of the study in subgroups of patients with the highest and lowest progression rates of SVD.

4. A panel of circulating inflammatory markers is measured in serum using the Olink proteomics platform (Olink Proteomics AB, Uppsala, Sweden).

At two-year follow-up, blood will be collected in EDTA tubes and processed according to manufacturer's instructions. Plasma will be stored at -80°C for future analysis of circulating inflammatory and coagulation markers.

For peer review only - http://bmjopen.bmj.com/site/about/guidelines.xhtml

2.3.4. Neuropsychological assessment

At baseline and two-year follow-up, participants undergo cognitive assessment using a test battery designed to capture characteristic cognitive impairment in SVD (**Supplementary Table 2**). Tests have been selected that are validated and normed for use in both countries and languages (English, Dutch); for a more extensive description of each test see Lezak et al. (2012).[27] Premorbid intelligence is assessed using the UK-developed National Adult Reading Test (NART-Revised) in Cambridge, and its Dutch equivalent (Dutch Adult Reading Test; DART) in Nijmegen. Processing speed is measured using the Symbol Digit Modalities Test (Written) and the Trail Making Test A. Working memory is measured using the forward and backward conditions of the Digit Span subtest of the Wechsler Adult Intelligence Scale – Fourth Edition (WAIS-IV). Executive function is assessed using the Trail Making Test B and the Brixton Spatial Anticipation Test. Visuospatial ability is evaluated using the Rey-Osterrieth Complex Figure Test (RCFT) - Copy trial. Memory is measured using the RCFT-Recall trial and the Story Recall subtest from the Rivermead Behavioural Memory Test (RBMT-3). The results of all tests will be converted to age-, education- and/or sex-adjusted standard scores using published country-specific normative data.

Self-administered questionnaires to assess psychological symptoms and social functioning are performed at the beginning of study visits to avoid responses being impacted by tiredness and affective changes from the clinical interview and cognitive tests. Depressive symptoms are assessed using the 30-item Geriatric Depression Scale (GDS).[28] Apathy is evaluated using the Apathy Evaluation Scale.[29] Fatigue is assessed using the Fatigue Severity Scale (FSS).[30] Social cognition is assessed using the Social Norms Questionnaire (SNQ).[31]

2.3.5. Gait assessment

Gait assessment is carried out at baseline and two-year follow-up using the Timed Up and Go (TUG) test and the 6-metre walk test. The TUG assesses gait performance and balance and is measured as the time (seconds) it takes a participant to rise from a chair, walk 3 metres, turn around, walk back to the chair and sit back down. The 6-metre walk test determines gait speed (metres/seconds). Participants have to walk a distance of 10 metres. Gait speed is assessed over a 6-metre distance by timing the participant from the 2-metre point to the 8-metre point. Participants are instructed to walk at a comfortable pace during both tests. The use of walking aids is recorded, if applicable. Both tests include a practice trial, followed by three timed trials which are averaged.

For peer review only - http://bmjopen.bmj.com/site/about/guidelines.xhtml

2.4. Data analysis

Peak height of the MD histogram was selected as the primary endpoint, given our past demonstration that it is the most sensitive DTI marker for SVD progression.[43] Based on longitudinal MRI data from the SCANS study, the yearly rate of change of MD peak height was -3.72×10^{-4} mm^2/s (SD 3.12×10^{-5}), which corresponds to an annual percentage change of -2.44%.[43] This would equate to a change of -4.88% over the two-year follow-up period of the INSVD study.

Our primary analysis will test whether baseline measures of inflammatory and immune markers predict SVD progression as measured by MD peak height. We assume a linear relationship between baseline levels of coagulation and inflammatory markers and the change in MD peak height. Therefore, we will run linear regression models and adjust for age, sex, education and other conventional MRI markers of SVD, where appropriate.

As our secondary objectives, we will assess (1) the relationship between inflammatory and immune markers and BBB permeability, both globally (cortical and subcortical volumes) and regionally (hotspot volumes), and (2) whether baseline levels of inflammatory and immune markers are associated with other imaging parameters, as well as cognitive and clinical outcomes. Similar to our primary analyses, we will conduct linear regression modelling to test these associations.

3. PATIENT AND PUBLIC INVOLVEMENT

This research has been informed through consultation with external stakeholders, including patients and their families, both in the UK and Netherlands. In joint initiatives with the Stroke Association (UK), SVD and cognitive decline have been identified as major priorities for patients with stroke. In a joint funder working group attended by both patients and their families, funders, and health professionals, research into the mechanisms underlying SVD was identified as a top priority. Study protocols were developed in partnership with local stroke support groups who advised on the study and logistics of implementation. To ensure that participants understand the study clearly before enrolment, the Participant Information Sheet was reviewed by two patient volunteers, whose comments were incorporated into the final document.

For peer review only - http://bmjopen.bmj.com/site/about/guidelines.xhtml

4. ETHICS AND DISSEMINATION

The study is conducted in accordance with the principles of the Declaration of Helsinki, the conditions and principles of Good Clinical Practice, and the applicable local regulatory requirements and laws. The project is funded by a joint grant from the British Heart Foundation and Dutch Heart Foundation (ref: SP/F/22/150028), and jointly sponsored by the Cambridge University Hospitals Foundation NHS Trust and the University of Cambridge in the UK, and the Radboud University Medical Centre in the Netherlands. Any participant-derived benefits resulting from this research, such as new insights into disease mechanisms or possible novel therapies, will be disseminated to study participants at patient groups and to members of the public through standardised communication platforms, including research journals and disease-specific national patient websites.

This study protocol has been registered on ClinicalTrials.gov (NCT05746221) and received ethical approval from the local ethics board (UK: East of England - Cambridge Central Research Ethics Committee (REC) ref: 22/EE/00141, Integrated Research Application System (IRAS) ID: 312747. NL: Medical Research Ethics Committee (MREC) Oost-Nederland, file number: 2022-13623, NL-number: NL80258.091.22).

5. CONCLUSION

Findings from this multicentre prospective cohort study could shed light on the role of immune (dys)regulation in SVD, potentially identifying specific components of the immune response relating to disease progression which could help identify new therapeutic targets and the development/repurposing of drugs to reduce or prevent SVD progression and vascular dementia.

For peer review only

For peer review only - http://bmjopen.bmj.com/site/about/guidelines.xhtml

REFERENCES

1 Wardlaw JM, Smith C, Dichgans M. Mechanisms underlying sporadic cerebral small vessel disease: insights from neuroimaging. *Lancet Neurology*. 2013;12:483–97.

2 Sachdev P, Kalaria R, O'Brien J, *et al.* Diagnostic criteria for vascular cognitive disorders: A VASCOG statement. *Alzheimer Dis Assoc Disord*. 2014;28:206–18.

3 Gorelick PB, Scuteri A, Black SE, *et al.* Vascular contributions to cognitive impairment and dementia: A statement for healthcare professionals from the American Heart Association/American Stroke Association. *Stroke*. 2011;42:2672–713.

4 Markus HS, Erik de Leeuw F. Cerebral small vessel disease: Recent advances and future directions. *International Journal of Stroke*. 2023;18:4–14.

5 Williamson JD, Pajewski NM, Auchus AP, *et al.* Effect of Intensive vs Standard Blood Pressure Control on Probable Dementia: A Randomized Clinical Trial. *JAMA*. 2019;321:553–61.

6 Wardlaw JM, Smith C, Dichgans M. Small vessel disease: mechanisms and clinical implications. *Lancet Neurol*. Published Online First: 2019. doi: 10.1016/S1474-4422(19)30079-1

7 Low A, Mak E, Rowe JB, *et al.* Inflammation and cerebral small vessel disease: A systematic review. *Ageing Res Rev*. 2019;53:100916.

8 Low A, Mak E, Malpetti M, *et al.* In vivo neuroinflammation and cerebral small vessel disease in mild cognitive impairment and Alzheimer's disease. *J Neurol Neurosurg Psychiatry*. 2021;92:45–52.

9 Walsh J, Tozer DJ, Sari H, *et al.* Microglial activation and blood–brain barrier permeability in cerebral small vessel disease. *Brain*. 2021;144:1361–71.

10 Simpson JE, Ince PG, Higham CE, *et al.* Microglial activation in white matter lesions and nonlesional white matter of ageing brains. *Neuropathol Appl Neurobiol*. 2007;33:670–83.

11 Noz MP, Ter Telgte A, Wiegertjes K, *et al.* Trained immunity characteristics are associated with progressive cerebral small vessel disease. *Stroke*. 2018;49:2910–7.

12 Noz MP, Ter Telgte A, Wiegertjes K, *et al.* Pro-inflammatory Monocyte Phenotype During Acute Progression of Cerebral Small Vessel Disease. *Front Cardiovasc Med*. 2021;8:639361.

13 Riksen NP, Bekkering S, Mulder WJM, *et al.* Trained immunity in atherosclerotic cardiovascular disease. *Nature Reviews Cardiology 2023*. 2023;1–13.

14 Wendeln A-C, Degenhardt K, Kaurani L, *et al.* Innate immune memory in the brain shapes neurological disease hallmarks. *Nature*. 2018;556:332–8.

For peer review only - http://bmjopen.bmj.com/site/about/guidelines.xhtml

15 Tomimoto H, Akiguchi I, Suenaga T, *et al.* Alterations of the blood-brain barrier and glial cells in white-matter lesions in cerebrovascular and Alzheimer's disease patients. *Stroke*. 1996;27:2069–74.

16 Wardlaw JM, Doubal F, Armitage P, *et al.* Lacunar stroke is associated with diffuse blood-brain barrier dysfunction. *Ann Neurol*. 2009;65:194–202.

17 Taheri S, Gasparovic C, Huisa BN, *et al.* Blood-Brain Barrier Permeability Abnormalities in Vascular Cognitive Impairment. *Stroke*. 2011;42:2158.

18 Traylor M, Persyn E, Tomppo L, *et al.* Genetic basis of lacunar stroke: a pooled analysis of individual patient data and genome-wide association studies. *Lancet Neurol*. 2021;20:351–61.

19 Topakian R, Barrick TR, Howe FA, *et al.* Blood–brain barrier permeability is increased in normal-appearing white matter in patients with lacunar stroke and leucoaraiosis. *J Neurol Neurosurg Psychiatry*. 2010;81:192–7.

20 Jalal FY, Yang Y, Thompson JF, *et al.* Hypoxia-induced neuroinflammatory white-matter injury reduced by minocycline in SHR/SP. *Journal of Cerebral Blood Flow and Metabolism*. 2015;35:1145–53.

21 Van Norden AGW, De Laat KF, Van Dijk EJ, *et al.* Diffusion tensor imaging and cognition in cerebral small vessel disease The RUN DMC study☆. *BBA - Molecular Basis of Disease*. 2012;1822:401–7.

22 Wardlaw JM, Smith EE, Biessels GJ, *et al.* Neuroimaging standards for research into small vessel disease and its contribution to ageing and neurodegeneration. *Lancet Neurol*. 2013;12:822–38.

23 Duering M, Biessels GJ, Brodtmann A, *et al.* Neuroimaging standards for research into small vessel disease—advances since 2013. *Lancet Neurol*. 2023;0. doi: 10.1016/S1474-4422(23)00131-X

24 van Norden AGW, de Laat KF, Gons RAR, *et al.* Causes and consequences of cerebral small vessel disease. The RUN DMC study: A prospective cohort study. Study rationale and protocol. *BMC Neurol*. 2011;11:1–8.

25 Staszewski J, Piusińska-Macoch R, Brodacki B, *et al.* IL-6, PF-4, sCD40 L, and homocysteine are associated with the radiological progression of cerebral small-vessel disease: A 2-year follow-up study. *Clin Interv Aging*. 2018;13:1135–41.

26 Bonita R, Beaglehole R. Modification of Rankin Scale: Recovery of motor function after stroke. *Stroke*. 1988;19:1497–500.

For peer review only - http://bmjopen.bmj.com/site/about/guidelines.xhtml

27 Lezak MD, Howieson DB, Bigler ED, *et al.* *Neuropsychological assessment*. 5th ed. New York, NY, US: Oxford University Press 2012.

28 Yesavage JA, Brink TL, Rose TL, *et al.* Development and validation of a geriatric depression screening scale: A preliminary report. *J Psychiatr Res*. 1982;17:37–49.

29 Marin RS, Biedrzycki RC, Firinciogullari S. Reliability and validity of the apathy evaluation scale. *Psychiatry Res*. 1991;38:143–62.

30 Krupp LB, Larocca NG, Muir Nash J, *et al.* The Fatigue Severity Scale: Application to Patients With Multiple Sclerosis and Systemic Lupus Erythematosus. *Arch Neurol*. 1989;46:1121–3.

31 Ganguli M, Sun Z, Mcdade E, *et al.* That's Inappropriate! Social Norms in an Older Population-based Cohort. *Alzheimer Dis Assoc Disord*. 2018;32:150–5.

32 Lippi G, Daves M, Mattiuzzi C. Interference of medical contrast media on laboratory testing. *Biochem Med (Zagreb)*. 2014;24:80–8.

33 Scheiermann C, Kunisaki Y, Frenette PS. Circadian control of the immune system. *Nature Reviews Immunology 2013 13:3*. 2013;13:190–8.

34 Moeller S, Yacoub E, Olman CA, *et al.* Multiband multislice GE-EPI at 7 tesla, with 16-fold acceleration using partial parallel imaging with application to high spatial and temporal whole-brain fMRI. *Magn Reson Med*. 2010;63:1144–53.

35 Xu J, Moeller S, Auerbach EJ, *et al.* Evaluation of slice accelerations using multiband echo planar imaging at 3T. *Neuroimage*. 2013;83:991–1001.

36 Feinberg DA, Moeller S, Smith SM, *et al.* Multiplexed Echo Planar Imaging for Sub-Second Whole Brain FMRI and Fast Diffusion Imaging. *PLoS One*. 2010;5:e15710-.

37 Smith SM, Jenkinson M, Woolrich MW, *et al.* Advances in functional and structural MR image analysis and implementation as FSL. *Neuroimage*. 2004;23:S208–19.

38 Baykara E, Gesierich B, Adam R, *et al.* A Novel Imaging Marker for Small Vessel Disease Based on Skeletonization of White Matter Tracts and Diffusion Histograms. *Ann Neurol*. 2016;80:581–92.

39 Patlak CS, Blasberg RG. Graphical evaluation of blood-to-brain transfer constants from multiple-time uptake data. Generalizations. *Journal of Cerebral Blood Flow and Metabolism*. 1985;5:584–90.

40 Janssen E, ter Telgte A, Verburgt E, *et al.* The Hyperintense study: Assessing the effects of induced blood pressure increase and decrease on MRI markers of cerebral small vessel disease: Study rationale and protocol. *Eur Stroke J*. 2022;7:331–8.

For peer review only

For peer review only - http://bmjopen.bmj.com/site/about/guidelines.xhtml

41 Fazekas F. Magnetic Resonance Signal Abnormalities in Asymptomatic Individuals: Their Incidence and Functional Correlates. *Eur Neurol*. 1989;29:164–8.

42 Potter GM, Chappell FM, Morris Z, *et al.* Cerebral perivascular spaces visible on magnetic resonance imaging: development of a qualitative rating scale and its observer reliability. *Cerebrovasc Dis*. 2015;39:224–31.

43 Zeestraten EA, Benjamin P, Lambert C, *et al.* Application of Diffusion Tensor Imaging Parameters to Detect Change in Longitudinal Studies in Cerebral Small Vessel Disease. *PLoS One*. 2016;11:e0147836.

For peer review only

For peer review only - http://bmjopen.bmj.com/site/about/guidelines.xhtml

DECLARATIONS

Author statement: AL and SvW set up the study at their respective study sites, coordinated recruitment and data collection, and drafted the manuscript. LC and RW contributed to participant recruitment and data collection. RPCK and RM provided expertise on neuropsychological assessments. ZM, NPR and MN provided expertise on biofluid laboratory analysis, and supervised the processing of blood samples. MCM, DJT, AMT, and AvdK contributed their expertise in neuroimaging. ZM, NPR, HSM, and F-EdL are the principal investigators, secured funding and designed the overall study. All authors reviewed and revised the manuscript for intellectual content and approved the final manuscript.

Funding tatement: This work was supported by a joint grant from the British Heart Foundation and Dutch Heart Foundation (ref: SP/F/22/150028), and jointly sponsored by the Cambridge University Hospitals Foundation NHS Trust and the University of Cambridge in the UK, and the Radboud University Medical Centre in the Netherlands. Infrastructural support was provided by Cambridge British Heart Foundation Centre of Research Excellence (RE/18/1/34212) and Cambridge University Hospitals NIHR Biomedical Research Centre (BRC-1215-20014).

Acknowledgements: The University of Minnesota Center for Magnetic Resonance Research provided us with the pulse sequence software for DTI, which is gratefully acknowledged. We thank our research volunteers for their participation.

Competing interests statement: The authors declare no conflicts of interest.

For peer review only

LIST OF FIGURES

Figure 1. Overview of study components.

Figure 2. Study flowchart; based on planned sample sizes.

For peer review only

For peer review only - http://bmjopen.bmj.com/site/about/guidelines.xhtml

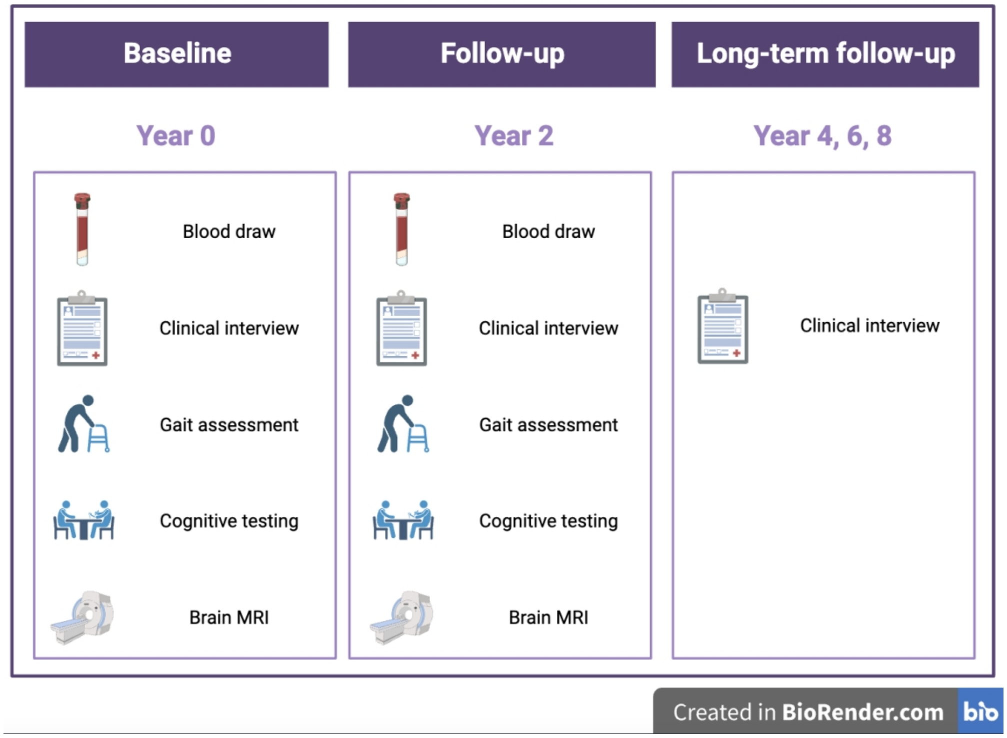

Figure 1. Overview of study components.

262x194mm (144 x 144 DPI)

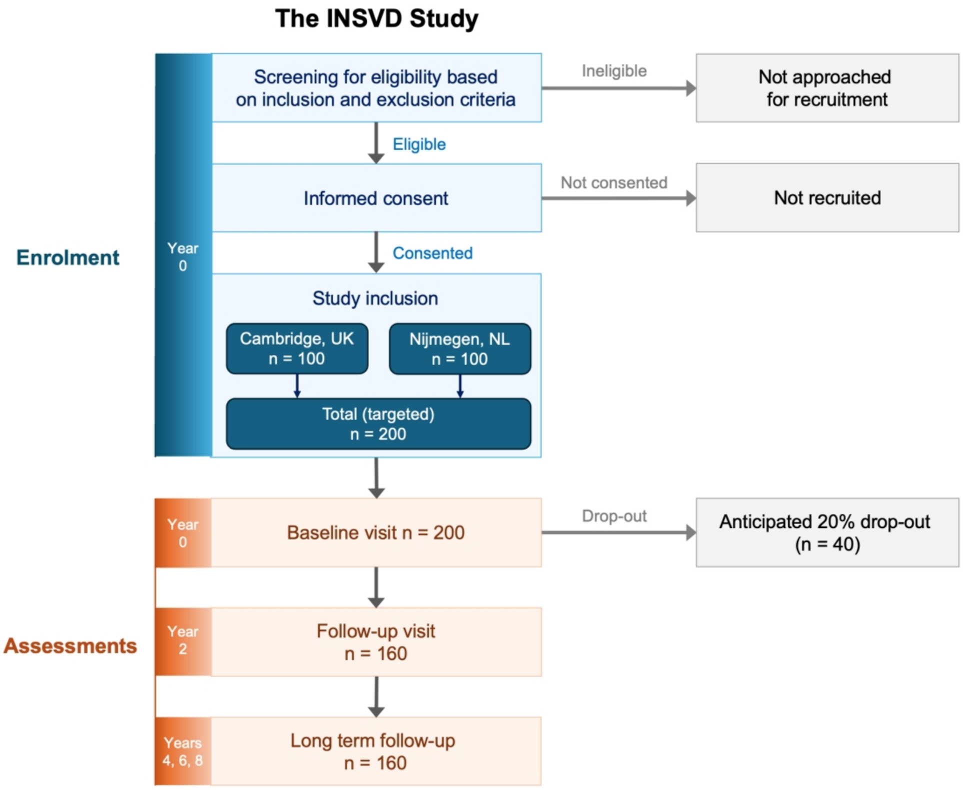

Figure 2. Study flowchart; based on planned sample sizes.

338x280mm (150 x 150 DPI)

Supplementary Table 1. Neuropsychological assessment measures

**Measured construct**	**Instrument**	**Mode of administration**	**Type of measure**
Premorbid intelligence	National/Dutch Adult Reading Test	Verbal	# correctly pronounced
Processing speed	Trail Making Test A	Written	time, # errors
	Symbol Digit Modalities Test	Written	# completed in 90s
Working memory	Digit Span Forwards and Backwards	Verbal	# correct trials
	Story Recall – Immediate Recall	Verbal	# correct elements recalled
Executive function	Trail Making Test B	Verbal	time, # errors
	Brixton Spatial Anticipation Test	Verbal	# errors
Visuospatial ability	Rey-Osterrieth Complex Figure Copy	Written	# correct elements drawn
Memory	Story Recall – Delayed Recall	Verbal	# correct elements recalled
	Rey-Osterrieth Complex Figure Recall	Written	# correct elements recalled
Depression	Geriatric Depression Scale (30-item version)	Self-administered questionnaire	Yes/No
Apathy	Apathy Evaluation Scale – Self	Self-administered questionnaire	4-point scale
	Apathy Evaluation Scale – Informant	Informant-rated questionnaire	4-point scale
Fatigue	Fatigue Severity Scale	Self-administered questionnaire	7-point scale
Social cognition	Social Norms Questionnaire	Self-administered questionnaire	Yes/No

For peer review only

Supplementary Table 2. MRI acquisition parameters.

	T1		FLAIR		T2* gradient echo		DTI		IVIM		DCE	
					SWI	QSM						
**Study site**	C	N	C	N	C	N	C	N	C	N	C	N
**Sequence**	3D MPRAGE		3D SPACE		3D SPGR		Single-shot spin-echo EPI		NA	Single-shot spin-echo EPI	3D SPGR	
**TR,** ms	2300		6000	5000	29	40	2433		NA	3386	15	
**TE,** ms	2.98		383	394	20	3.5, 8.6, 13.7, 18.9, 24.0, 29.2, 34.3	75.6		NA	60.6	1.75	
**TI,** ms	900		2100	1800	NA	NA	NA		NA	NA	NA	
**Flip angle**	9°		multiple	multiple	15°	15°	80°		NA	90°	5°, 10°, 15°, 21°, 26°	
**Fat suppression**	None		None	None	None	WEX	FatSat		NA	FatSat	None	
**FOV** mm^2	256×248		256×256	250×250	256×256	256×256	192×192		NA	212×212	240×240	
**Voxel size** mm^3	1×1×1		1×1×1	0.5×0.5×1	0.5×0.5×2	1×1×1	1.7×1.7×1.8		NA	2×2×2	1.9×1.9×4.0	
**Slices**	176		192	176	72	176	76		NA	74	16	
**Slice orientation**	SAG		SAG	SAG	TRA	SAG	TRA		NA	TRA	TRA	
**Diffusion b-values** s/mm^2	NA		NA	NA	NA	NA	0, 300, 1000, 2000		NA	0, 5, 10, 15, 20, 30, 40, 50, 60, 100, 200, 400, 600, 800, 1000	NA	
**Diffusion gradient directions**	NA		NA	NA	NA	NA	6, 8, 30, 60		NA	6	NA	
**Parallel imaging accel. Factor**	2	3	2	2	2	6	NA		NA	2	NA	
**Multiband accel. Factor**	NA		NA	NA	NA	NA	4		NA	2	NA	
**Acquisition time,** mm:ss	05:12	03:01	05:44	04:42	04:35	05:06	04:28		NA	05:39	21:12	
**Temporal res.** mm:ss	NA		NA	NA	NA	NA	NA		NA	NA	02:39	

Abbreviations: C = Cambridge, DCE = dynamic contrast-enhanced MRI, DTI = diffusion tensor imaging (multi-shell with 98 gradient directions and 6 b=0 values), *FLAIR = fluid-attenuated inversion recovery, FOV = field of view, GRAPPA = GeneRalized Autocalibrating Partial Parallel Acquisition, IVIM = Intravoxel Incoherent Motion, N = Nijmegen, SAG = sagittal, SWI = susceptibility-weighted imaging, TI = inversion time, TE = echo time, TR = repetition time, TRA = transversal.*

Reporting checklist for cohort study.

Based on the STROBE cohort guidelines.

Instructions to authors

Complete this checklist by entering the page numbers from your manuscript where readers will find each of the items listed below.

Your article may not currently address all the items on the checklist. Please modify your text to include the missing information. If you are certain that an item does not apply, please write "n/a" and provide a short explanation.

Upload your completed checklist as an extra file when you submit to a journal.

In your methods section, say that you used the STROBE cohortreporting guidelines, and cite them as:

von Elm E, Altman DG, Egger M, Pocock SJ, Gotzsche PC, Vandenbroucke JP. The Strengthening the Reporting of Observational Studies in Epidemiology (STROBE) Statement: guidelines for reporting observational studies.

		Reporting Item	Page Number
**Title and abstract**			
Title	#1a	Indicate the study's design with a commonly used term in the title or the abstract	1
Abstract	#1b	Provide in the abstract an informative and balanced summary of what was done and what was found	2
**Introduction**			
Background / rationale	#2	Explain the scientific background and rationale for the investigation being reported	4
Objectives	#3	State specific objectives, including any prespecified hypotheses	5
**Methods**			

For peer review only

Study design	#4	Present key elements of study design early in the paper	6
Setting	#5	Describe the setting, locations, and relevant dates, including periods of recruitment, exposure, follow-up, and data collection	6
Eligibility criteria	#6a	Give the eligibility criteria, and the sources and methods of selection of participants. Describe methods of follow-up.	6
Eligibility criteria	#6b	For matched studies, give matching criteria and number of exposed and unexposed	na
Variables	#7	Clearly define all outcomes, exposures, predictors, potential confounders, and effect modifiers. Give diagnostic criteria, if applicable	8-11
Data sources / measurement	#8	For each variable of interest give sources of data and details of methods of assessment (measurement). Describe comparability of assessment methods if there is more than one group. Give information separately for for exposed and unexposed groups if applicable.	8-11
Bias	#9	Describe any efforts to address potential sources of bias	7,9
Study size	#10	Explain how the study size was arrived at	7
Quantitative variables	#11	Explain how quantitative variables were handled in the analyses. If applicable, describe which groupings were chosen, and why	12
Statistical methods	#12a	Describe all statistical methods, including those used to control for confounding	12
Statistical methods	#12b	Describe any methods used to examine subgroups and interactions	10
Statistical methods	#12c	Explain how missing data were addressed	7
Statistical methods	#12d	If applicable, explain how loss to follow-up was addressed	7
Statistical methods	#12e	Describe any sensitivity analyses	12

For peer review only

Results

Participants	#13a	Report numbers of individuals at each stage of study—eg numbers potentially eligible, examined for eligibility, confirmed eligible, included in the study, completing follow-up, and analysed. Give information separately for for exposed and unexposed groups if applicable.	7
Participants	#13b	Give reasons for non-participation at each stage	na
Participants	#13c	Consider use of a flow diagram	na
Descriptive data	#14a	Give characteristics of study participants (eg demographic, clinical, social) and information on exposures and potential confounders. Give information separately for exposed and unexposed groups if applicable.	na
Descriptive data	#14b	Indicate number of participants with missing data for each variable of interest	na
Descriptive data	#14c	Summarise follow-up time (eg, average and total amount)	6,7
Outcome data	#15	Report numbers of outcome events or summary measures over time. Give information separately for exposed and unexposed groups if applicable.	na
Main results	#16a	Give unadjusted estimates and, if applicable, confounder-adjusted estimates and their precision (eg, 95% confidence interval). Make clear which confounders were adjusted for and why they were included	na
Main results	#16b	Report category boundaries when continuous variables were categorized	9
Main results	#16c	If relevant, consider translating estimates of relative risk into absolute risk for a meaningful time period	na

For peer review only

Other analyses	#17	Report other analyses done—eg analyses of subgroups and interactions, and sensitivity analyses	12
**Discussion**			
Key results	#18	Summarise key results with reference to study objectives	na
Limitations	#19	Discuss limitations of the study, taking into account sources of potential bias or imprecision. Discuss both direction and magnitude of any potential bias.	3
Interpretation	#20	Give a cautious overall interpretation considering objectives, limitations, multiplicity of analyses, results from similar studies, and other relevant evidence.	na
Generalisability	#21	Discuss the generalisability (external validity) of the study results	na
**Other Information**			
Funding	#22	Give the source of funding and the role of the funders for the present study and, if applicable, for the original study on which the present article is based	2,13

None The STROBE checklist is distributed under the terms of the Creative Commons Attribution License CC-BY. This checklist can be completed online using https://www.goodreports.org/, a tool made by the EQUATOR Network in collaboration with Penelope.ai

For peer review only

BMJ Open

Immune regulation and blood-brain barrier permeability in cerebral small vessel disease – study protocol of the INflammation and Small Vessel Disease (INSVD) study, a multicentre prospective cohort study

Journal:	*BMJ Open*
Manuscript ID	bmjopen-2024-084303.R1
Article Type:	Protocol
Date Submitted by the Author:	08-Feb-2024

Complete List of Authors:	Low, Audrey; University of Cambridge, Department of Clinical Neurosciences<br>van Winden, Sanne; Radboudumc, Department of Neurology; Radboud University Donders Institute for Brain Cognition and Behaviour<br>Cai, Lupei; University of Cambridge, Department of Clinical Neurosciences<br>Kessels, Roy; Radboud University Donders Institute for Brain Cognition and Behaviour; Vincent Van Gogh Instituut<br>Maas, Marnix C.; Radboudumc, Department of Radiology and Nuclear Medicine<br>Morris, Robin; King's College London, Institute of Psychiatry, Psychology and Neuroscience (IoPPN)<br>Nus, Meritxell; University of Cambridge, Division of Cardiovascular Medicine, Department of Medicine<br>Tozer, Daniel; University of Cambridge, Department of Clinical Neurosciences<br>Tuladhar, Anil; Radboudumc, Department of Neurology; Radboud University Donders Institute for Brain Cognition and Behaviour<br>van der Kolk, Anja; Radboudumc, Department of Radiology and Nuclear Medicine<br>Wolters, Rowan; Radboudumc, Department of Neurology<br>Mallat, Ziad; University of Cambridge Medicine, The Victor Phillip Dahdaleh Heart and Lung Research Institute, Section of Cardiorespiratory Medicine, Department of Medicine<br>Riksen, Niels; Radboudumc, Department of Internal Medicine<br>Markus, Hugh; University of Cambridge, Department of Clinical Neurosciences<br>de Leeuw, Frank-Erik ; Radboudumc, Department of Neurology

**Primary Subject Heading**:	Neurology
Secondary Subject Heading:	Immunology (including allergy), Cardiovascular medicine
Keywords:	Aged, Stroke < NEUROLOGY, IMMUNOLOGY, Magnetic resonance imaging < RADIOLOGY & IMAGING, Old age psychiatry < PSYCHIATRY

Immune regulation and blood-brain barrier permeability in cerebral small vessel disease – study protocol of the INflammation and Small Vessel Disease (INSVD) study, a multicentre prospective cohort study

Audrey Low[1,*], Sanne van Winden[2,3,*], Lupei Cai[1], Roy P.C. Kessels[3,4,5], Marnix C. Maas[6], Robin Morris[7], Meritxell Nus[8], Daniel J. Tozer[1], Anil M. Tuladhar[2,3], Anja van der Kolk[6], Rowan Wolters[2], Ziad Mallat[8], Niels P. Riksen[9], Hugh S. Markus[1,†], Frank-Erik de Leeuw[2,3,†]

Affiliations
[1] Stroke Research Group, Department of Clinical Neurosciences, University of Cambridge, UK
[2] Department of Neurology, Radboud Research Institute for Medical Innovation, Radboud University Medical Centre, Nijmegen, The Netherlands
[3] Donders Institute for Brain, Cognition, and Behaviour, Radboud University, Nijmegen, The Netherlands
[4] Department of Medical Psychology and Radboudumc Alzheimer Centre, Radboud University Medical Centre, Nijmegen, The Netherlands
[5] Vincent van Gogh Institute for Psychiatry, Venray, The Netherlands
[6] Department of Medical Imaging, Radboud University Medical Centre, Nijmegen, The Netherlands
[7] Department of Psychology, King's College London, Institute of Psychiatry, Psychology and Neuroscience, UK
[8] The Victor Phillip Dahdaleh Heart and Lung Research Institute, Section of Cardiorespiratory Medicine, Department of Medicine, University of Cambridge, UK
[9] Department of Internal Medicine, Radboud University Medical Centre, Nijmegen, The Netherlands

* joint first authors
† joint senior authors

Corresponding Author
Audrey Low
Department of Clinical Neurosciences, Neurology Unit, University of Cambridge
School of Clinical Medicine, R3, Box 83, Cambridge Biomedical Campus
Cambridge, Cambridgeshire, United Kingdom, CB2 0SP
Email: al927@cam.ac.uk

MeSH Keywords: Aged, Stroke, Immunology, Magnetic Resonance Imaging, Old age psychiatry

Word Count: 3152

For peer review only - http://bmjopen.bmj.com/site/about/guidelines.xhtml

ABSTRACT

Introduction

The INflammation and Small Vessel Disease (INSVD) study aims to investigate whether peripheral inflammation, immune (dys)regulation and blood-brain barrier (BBB) permeability relate to disease progression in cerebral small vessel disease (SVD). This research aims to pinpoint specific components of the immune response in SVD relating to disease progression. This could identify biomarkers of SVD progression, as well as potential therapeutic targets to inform the development and repurposing of drugs to reduce or prevent SVD, cognitive decline, and vascular dementia.

Methods and analysis

INSVD is a prospective observational multicentre cohort study in individuals with symptomatic SVD. This longitudinal study combines comprehensive immunophenotyping of the peripheral blood immune compartment with advanced neuroimaging markers of SVD and BBB permeability. The main SVD marker of interest is white matter microstructure as determined by diffusion tensor imaging (DTI), a valuable marker of disease progression owing to its sensitivity to early alterations to white matter integrity. The research is being conducted in two sites – in the United Kingdom (Cambridge) and the Netherlands (Nijmegen) – with each site recruiting 100 participants (total n=200). Participants undergo clinical and cognitive assessments, blood draws, and brain MRI at baseline and two-year follow-up.

Ethics and dissemination

This study received ethical approval from the local ethics board (UK: East of England - Cambridge Central Research Ethics Committee (REC) ref: 22/EE/00141, Integrated Research Application System (IRAS) ID: 312747. NL: Medical Research Ethics Committee (MREC) Oost-Nederland, ref: 2022-13623, NL-number: NL80258.091.22). Written informed consent was obtained from all subjects before the study. Any participant-derived benefits resulting from this research, such as new insights into disease mechanisms or possible novel therapies, will be disseminated to study participants, patient groups, and members of the public.

Fundings and public registration

The INSVD study is funded by a joint grant from the British Heart Foundation and Dutch Heart Foundation (SP/F/22/150028) and has been registered on ClinicalTrials.gov (NCT05746221).

For peer review only - http://bmjopen.bmj.com/site/about/guidelines.xhtml

ARTICLE SUMMARY

Strengths and limitations of this study

- Strength: Deep immunophenotyping of the peripheral blood immune compartment using state-of-the-art (epi)genetic, transcriptomic and proteomic techniques and functional assays
- Strength: Longitudinal cohort study design to track disease progression
- Strength: Advanced neuroimaging techniques to measure and map blood-brain barrier integrity across the brain
- Strength: Multicentre study with comparable neuroimaging protocols on identical 3T MRI scanners in both sites
- Limitation: Deep immunophenotyping only performed at baseline, thus this study is not able to assess whether *changes* in immune cell phenotype are predictive of disease progression

For peer review only

1. INTRODUCTION

Cerebral small vessel disease (SVD) is a common condition in older adults characterised by injury to the small penetrating vessels of the brain, and accounts for a quarter of all strokes, both intracerebral haemorrhages and lacunar strokes.[1] As well as resulting in vascular cognitive impairment (VCI),[2,3] SVD is the major vascular pathology contributing to dementia.[4] At present, there are no disease-modifying treatments for the resulting VCI, and treatments to reduce SVD are limited to controlling vascular risk factors like hypertension.[5] This lack of treatment options is mainly due to an incomplete understanding of the underlying pathophysiology of SVD, although emerging evidence highlights candidate mechanisms such as inflammation and blood-brain barrier (BBB) dysfunction.[6]

The presence of inflammation and immune dysregulation in SVD has been revealed both by blood markers of inflammation,[7] and by 'inflammation' in the central nervous system, observed through neuroimaging (e.g., activated microglia in the brain)[8,9] and neuropathological examination.[10] Recent exploratory studies showed an association between the cytokine production capacity of circulating monocytes and SVD progression.[11,12] Innate immune cells can develop a persistent hyperinflammatory phenotype after brief stimulation by epigenetic reprogramming, which has been termed trained immunity.[13] This also occurs in microglia and can contribute to neuropathology.[14] We hypothesize that trained circulating and cerebral innate immune cells contribute to SVD progression. However, there remains much uncertainty about the underlying processes linking inflammation to SVD and, in particular, whether inflammation is causally related to disease progression.

Another closely related factor implicated in SVD pathogenesis is BBB permeability.[15–19] Impaired BBB function is evidenced by albumin leakage into the cerebrospinal fluid or the imaging of gadolinium contrast agent leakage into the brain parenchyma.[15,19] Normally, the BBB is selective of the blood components allowed to enter the central nervous system. In a dysfunctional state, however, BBB permeability is impaired, leading to the dysregulated leakage of substances into the brain, including harmful substances. Importantly, BBB dysfunction has been reported to be related to chronic unresolved inflammation and *in vivo* studies show a potential shared pathogenic pathway.[20] As for inflammation, however, it is uncertain whether BBB permeability plays a causal role in SVD pathogenesis, and how it relates to both systemic and central inflammation in patients.

The *INflammation and Small Vessel Disease* (INSVD) study has been established to address these questions; in particular, to understand the relationship between inflammation and BBB permeability, and

For peer review only - http://bmjopen.bmj.com/site/about/guidelines.xhtml

their potential relationship with SVD progression. INSVD is a longitudinal cohort study investigating whether systemic inflammation and immune (dys)function, and BBB permeability predict disease progression in SVD, with our primary SVD outcome measure being white matter microstructure as determined by diffusion tensor imaging (DTI), which is a valuable marker of disease progression owing to its sensitivity to early alterations to white matter integrity.[21]

Our overarching aim is to examine in detail the phenotype of circulating immune cells in patients with SVD to identify specific components of the dysregulated immune response which relate to disease progression. This could help identify biomarkers of disease progression and potential therapeutic targets for the development and repurposing of drugs to reduce SVD, VCI, and vascular dementia. Study objectives are as follows:

Primary objective

- To determine whether changes in immune cell function and phenotype identified in the systemic circulation predict disease progression in SVD, as measured by DTI-MRI

Secondary objectives

- To characterise the alteration in immune phenotype occurring in SVD and how these relate to clinical, neurocognitive and imaging parameters
- To determine the relationships between systemic immune changes in SVD and BBB leakage

We hypothesise that activation of the immune system plays a causal role in the progression of SVD, and that mediating processes resulting from this immune activation may involve BBB leakage and inflammation. This progressive damage then compromises structural and functional brain integrity, leading to progressive white matter damage and accelerated VCI.

For peer review only - http://bmjopen.bmj.com/site/about/guidelines.xhtml

2. METHODS AND ANALYSIS

2.1. Overview

The INSVD study is a prospective multicentre observational cohort study in patients with SVD. The research will be conducted at two sites – Cambridge (UK), and Nijmegen (The Netherlands) – each recruiting 100 participants (total n=200). At baseline and two-year follow-up, participants undergo clinical and cognitive assessments, blood draw, and brain MRI. Follow-up will be continued for the next six years to track incident clinical events, among others (**Figure 1**). Study recruitment began in August 2022 and study visits (baseline and two-year follow-up) are projected to be completed by August 2026.

[Figure 1 here]

2.2. Participants

2.2.1. Inclusion criteria

The study recruits individuals aged 40 and above with symptomatic SVD – this is defined as having a lacune on MRI according to the Standards for Reporting Vascular Changes on Neuroimaging (STRIVE) guidelines,[22,23] in addition to at least one of the three following clinical presentations: (a) clinical lacunar stroke syndrome; (b) symptoms of cognitive impairment presumed to be due to SVD; (c) gait/motor impairment presumed to be due to SVD. Baseline study visits are performed after a minimum interval of three months after participants' most recent stroke and at least one month after any systemic infection or vaccination.

2.2.2. Exclusion criteria

Potential participants are excluded if they have any MRI contraindication, or an estimated glomerular filtration rate (eGFR) <60ml/min/1.73m^2 (UK) or <30ml/min/1.73m^2 (Netherlands) within the past three months (Figure 2). If eGFR has not been assessed within the last three months, this is measured as part of the research procedure. Other exclusion criteria include strokes caused by factors other than SVD (e.g., cardioembolic source, carotid or vertebral artery stenosis >50%), another diagnosed chronic neurological condition (e.g., Alzheimer's disease, Parkinson's disease), myocardial infarction in the past year, vasculitis, autoimmune or autoinflammatory disease, treatment with immunomodulating drugs, known monogenic

For peer review only - http://bmjopen.bmj.com/site/about/guidelines.xhtml

causes of SVD, or limited life expectancy due to another illness or chronic condition which would make the two-year follow-up difficult (e.g., widespread malignancy).

[Figure 2 here]

2.2.3. Participant recruitment

At the Cambridge site, participants are screened and recruited from the clinical stroke service at Cambridge University Hospitals NHS Foundation Trust and patient registers of SVD patients enrolled in previous research studies. Screening is conducted through electronic hospital records, discussions with the patient about relevant medical history, and through the local MRI safety questionnaire. ECG and carotid artery duplex scans have been performed as part of ischemic stroke workup, and are used to rule out stroke causes other than SVD.

At the Nijmegen site, participants are recruited from the RUN DMC study cohort,[24] and the neurology outpatient clinic at Radboud University Medical Centre. Screening involves reviewing electronic hospital records and discussing medical history with the patient. ECG and carotid artery duplex scans are included as part of screening procedures, in case information on atrial fibrillation and large artery disease are not up to date or readily available from medical records.

2.2.4. Sample size calculation

Power calculations were based on previous findings demonstrating associations between circulating IL-6 and SVD progression,[25] and our pilot data showing a correlation between IL-6 production in peripheral blood mononuclear cells (PBMC; after ex vivo toll-like receptor-2 (TLR2) stimulation) and SVD progression (white matter hyperintensity (WMH) progression) of $r=0.22$.[11] To detect a correlation of $r=0.22$ at $\alpha=0.05$ with 80% power (two-tailed test with Fisher formula), 160 participants with evaluable datasets are needed. To account for dropouts and unusable data (e.g., failed MRI quality check) estimated at 20%, we are recruiting 200 participants.

For peer review only - http://bmjopen.bmj.com/site/about/guidelines.xhtml

2.3. Measures

2.3.1. Clinical assessment

Demographics, medical history, medication use, cardiovascular risk factors (e.g., diabetes, hypercholesterolaemia, hypertension), and behavioural risk factors (e.g., smoking, alcohol consumption) are recorded based on electronic medical records and by interviewing participants at baseline. Clinical details of the most recent stroke are assessed, where relevant. Blood pressure and heart rate are measured after the participant has been seated comfortably for at least five minutes. Three measurements are collected, and means are calculated as the average of the last two readings. Height and weight are recorded to determine body mass index. Global disability is assessed using the Modified Rankin Score (mRS).[26] At long-term follow-up visits, we record incident clinical events and changes in medication use and cardiovascular risk factors.

2.3.2. Neuroimaging

All participants undergo brain MRI, acquired at both sites on identical 3T scanners (MAGNETOM PrismaFit, Siemens Healthineers, Erlangen, Germany) with a 32-channel head coil. The acquisition protocol consists of the following sequences: three-dimensional (3D) T_1-weighted magnetization prepared rapid gradient echo (MPRAGE), 3D T_2-weighted fluid-attenuated inversion recovery (FLAIR), 3D T_2*-weighted susceptibility weighted imaging (SWI, Cambridge) or quantitative susceptibility mapping (QSM, Nijmegen), intravoxel incoherent motion imaging (IVIM, Nijmegen only), multi-shell diffusion-weighted imaging (DWI),[27–29] and dynamic contrast enhanced (DCE) imaging (**Supplementary Table 1**). DWI is acquired twice with opposing phase-encoding directions to enable correction for magnetic field and eddy current-induced geometric distortions. A B_0 field map and an additional b=0 s/mm^2 image with opposed phase-encoding polarity are acquired to correct for geometric distortions in IVIM imaging.

The study employs an optimised DCE-MRI protocol[17] which has been implemented in other studies conducted by the Cambridge research group.[9] This uses a fast T_1 mapping approach based on variable flip angle (VFA) spoiled gradient echo (SPGR) imaging, before and after the injection of a gadolinium-based contrast agent. Following one pre-contrast T_1 mapping acquisition, a 0.025mmol/kg dose of the gadolinium agent gadoterate meglumine (Dotarem®, Guerbet, Villepinte, France) is administered by a medically qualified staff member. Post-contrast T_1 mapping is started one minute after

For peer review only - http://bmjopen.bmj.com/site/about/guidelines.xhtml

administration and continued for approximately 20 minutes (7 time points) to map the signal changes that are used to calculate permeability.

T_1-weighted MPRAGE images will be processed using standard imaging processing pipelines (e.g., SIENAX) to obtain maps of grey matter, white matter, and cerebrospinal fluid. Image output will be visually inspected for misclassifications, and then used in conjunction with WMH masks to estimate brain and tissue volumes. Diffusion imaging data are pre-processed using the FMRIB Software Library (FSL)[30] and MRtrix3 (mrtrix.org). Specifically, we will perform denoising, Gibbs artefact removal and correction for head motion, eddy current distortions and susceptibility-induced distortions. Diffusion data will be analysed using voxel-by-voxel maps of standard diffusion parameters (e.g., MD, FA) to produce whole-tissue histograms. Additionally, Peak Width of Skeletonized Mean Diffusivity (PSMD)[31] will be derived from DTI. PSMD is calculated from tissue on a white matter skeleton and is a measure of the width of the MD histogram. For DCE analysis, T_1 maps are first computed based on the VFA SPGR images using the scanner software (MAPIT package, Siemens Healthineers), correcting for B_1 inhomogeneity using a separately acquired pre-contrast B_1 map. The T1 maps are spatially coregistered for motion correction, and pharmacokinetic analysis is performed on a voxel-by-voxel basis using the Patlak model,[32] yielding maps of K_{trans}, the blood-to-tissue transfer constant. The T_1 maps – and hence the K_{trans} maps – are also registered to the conventional images and used for analysis alongside manually delineated lesion masks. IVIM images will be processed and analysed as described previously.[33]

Participants will also be assessed on four SVD markers, namely WMH, lacunes, CMB, and PVS, according to the STRIVE-2 criteria.[23] All scans will be visually assessed by trained raters, blinded to all demographic and clinical information. WMH maps will be obtained from FLAIR images using an automated tool. Lesion maps will be reviewed by a single trained rater blinded to all clinical information and manually corrected for misclassifications. Haemorrhages and isolated lacunes will be excluded from the WMH mask. WMH volumes will be extracted from the finalised lesion masks and normalised by total intracranial volume. WMH will also be assessed semi-quantitatively through visual rating.[34] Periventricular and deep WMH will be rated separately. Lacunes and PVS will be identified manually using T_1-weighted and FLAIR images and delineated to produce lesion maps. PVS, CMB and lacunes will also be rated semi-quantitatively using validated scales where available.[35] PVS will be assessed separately in the basal ganglia and centrum semiovale to account for different underlying pathologies. CMB will be manually delineated using SWI (Cambridge) and QSM images (Nijmegen) to derive CMB lesion maps.

For peer review only - http://bmjopen.bmj.com/site/about/guidelines.xhtml

2.3.3. Blood samples

Non-fasting, venous blood samples are drawn at baseline and two-year follow-up by qualified clinical personnel. Blood withdrawal is performed before MRI to ensure that the injected gadolinium-based MRI contrast agent does not interfere with laboratory tests.[36] Blood samples are collected at approximately the same time of day for all participants to minimise circadian-related variation in inflammatory and coagulation markers.[37] At baseline, blood is collected in EDTA tubes for plasma, serum tubes for serum, and BD Vacutainer CPT tubes (BD Biosciences) for whole blood and PBMCs. Blood tubes are stored at room temperature and processed within 2 hours after collection for the following procedures:

1. PBMCs are isolated using sodium heparin CPT tubes following manufacturer recommendations and freshly stimulated for 24 hours with the TLR-4 agonist lipopolysaccharide (LPS), with the TLR-2 agonist Pam3Cys-Ser-(Lys)4, with LPS plus the inflammasome activator Nigericin, or with RPMI buffer as control. Supernatants are stored at -80°C for future analysis of cytokines and chemokines.

2. Comprehensive immunophenotyping of fresh whole blood is performed using Cytometry by Time-Of-Flight (CyTOF).

3. Isolated PBMCs are viably frozen to allow future single-cell RNA sequencing (scRNA-seq), Assay of Transposase Accessible Chromatin with high-throughput sequencing (ATAC-seq) and regulatory T cell (Treg) suppression assays. ScRNA-seq, ATAC-seq and Treg suppression assays will be performed after completion of the study in subgroups of patients with the highest and lowest progression rates of SVD.

4. A panel of circulating inflammatory markers is measured in serum using the Olink proteomics platform (Olink Proteomics AB, Uppsala, Sweden).

At two-year follow-up, blood will be collected in EDTA tubes and processed according to manufacturer's instructions. Plasma will be stored at -80°C for future analysis of circulating inflammatory and coagulation markers.

For peer review only

2.3.4. Neuropsychological assessment

At baseline and two-year follow-up, participants undergo cognitive assessment using a test battery designed to capture characteristic cognitive impairment in SVD (**Supplementary Table 2**). Tests have been selected that are validated and normed for use in both countries and languages (English, Dutch); for a more extensive description of each test see Lezak et al. (2012).[38] Premorbid intelligence is assessed using the UK-developed National Adult Reading Test (NART-Revised) in Cambridge, and its Dutch equivalent (Dutch Adult Reading Test; DART) in Nijmegen. Processing speed is measured using the Symbol Digit Modalities Test (Written) and the Trail Making Test A. Working memory is measured using the forward and backward conditions of the Digit Span subtest of the Wechsler Adult Intelligence Scale – Fourth Edition (WAIS-IV). Executive function is assessed using the Trail Making Test B and the Brixton Spatial Anticipation Test. Visuospatial ability is evaluated using the Rey-Osterrieth Complex Figure Test (RCFT) - Copy trial. Memory is measured using the RCFT-Recall trial and the Story Recall subtest from the Rivermead Behavioural Memory Test (RBMT-3). The results of all tests will be converted to age-, education- and/or sex-adjusted standard scores using published country-specific normative data.

Self-administered questionnaires to assess psychological symptoms and social functioning are performed at the beginning of study visits to avoid responses being impacted by tiredness and affective changes from the clinical interview and cognitive tests. Depressive symptoms are assessed using the 30-item Geriatric Depression Scale (GDS).[39] Apathy is evaluated using the Apathy Evaluation Scale.[40] Fatigue is assessed using the Fatigue Severity Scale (FSS).[41] Social cognition is assessed using the Social Norms Questionnaire (SNQ).[42]

2.3.5. Gait assessment

Gait assessment is carried out at baseline and two-year follow-up using the Timed Up and Go (TUG) test and the 6-metre walk test. The TUG assesses gait performance and balance and is measured as the time (seconds) it takes a participant to rise from a chair, walk 3 metres, turn around, walk back to the chair and sit back down. The 6-metre walk test determines gait speed (metres/seconds). Participants have to walk a distance of 10 metres. Gait speed is assessed over a 6-metre distance by timing the participant from the 2-metre point to the 8-metre point. Participants are instructed to walk at a comfortable pace during both tests. The use of walking aids is recorded, if applicable. Both tests include a practice trial, followed by three timed trials which are averaged.

For peer review only - http://bmjopen.bmj.com/site/about/guidelines.xhtml
For peer review only

2.4. Data analysis

Peak height of the MD histogram was selected as the primary endpoint, given our past demonstration that it is the most sensitive DTI marker for SVD progression.[43] Based on longitudinal MRI data from the SCANS study, the yearly rate of change of MD peak height was -3.72×10^{-4} mm^2/s (SD 3.12×10^{-5}), which corresponds to an annual percentage change of -2.44%.[43] This would equate to a change of -4.88% over the two-year follow-up period of the INSVD study.

Our primary analysis will test whether baseline measures of inflammatory and immune markers predict SVD progression as measured by MD peak height. We assume a linear relationship between baseline levels of coagulation and inflammatory markers and the change in MD peak height. Therefore, we will run linear regression models and adjust for age, sex, education and other conventional MRI markers of SVD, where appropriate.

As our secondary objectives, we will assess (1) the relationship between inflammatory and immune markers and BBB permeability, both globally (cortical and subcortical volumes) and regionally (hotspot volumes), and (2) whether baseline levels of inflammatory and immune markers are associated with other imaging parameters, as well as cognitive and clinical outcomes. Similar to our primary analyses, we will conduct linear regression modelling to test these associations.

3. PATIENT AND PUBLIC INVOLVEMENT

This research has been informed through consultation with external stakeholders, including patients and their families, both in the UK and Netherlands. In joint initiatives with the Stroke Association (UK), SVD and cognitive decline have been identified as major priorities for patients with stroke. In a joint funder working group attended by both patients and their families, funders, and health professionals, research into the mechanisms underlying SVD was identified as a top priority. Study protocols were developed in partnership with local stroke support groups who advised on the study and logistics of implementation. To ensure that participants understand the study clearly before enrolment, the Participant Information Sheet was reviewed by two patient volunteers, whose comments were incorporated into the final document.

For peer review only - http://bmjopen.bmj.com/site/about/guidelines.xhtml

4. ETHICS AND DISSEMINATION

The study is conducted in accordance with the principles of the Declaration of Helsinki, the conditions and principles of Good Clinical Practice, and the applicable local regulatory requirements and laws. The project is funded by a joint grant from the British Heart Foundation and Dutch Heart Foundation (ref: SP/F/22/150028), and jointly sponsored by the Cambridge University Hospitals Foundation NHS Trust and the University of Cambridge in the UK, and the Radboud University Medical Centre in the Netherlands. Any participant-derived benefits resulting from this research, such as new insights into disease mechanisms or possible novel therapies, will be disseminated to study participants at patient groups and to members of the public through standardised communication platforms, including research journals and disease-specific national patient websites.

This study protocol has been registered on ClinicalTrials.gov (NCT05746221) and received ethical approval from the local ethics board (UK: East of England - Cambridge Central Research Ethics Committee (REC) ref: 22/EE/00141, Integrated Research Application System (IRAS) ID: 312747. NL: Medical Research Ethics Committee (MREC) Oost-Nederland, file number: 2022-13623, NL-number: NL80258.091.22).

For peer review only - http://bmjopen.bmj.com/site/about/guidelines.xhtml

DECLARATIONS

Contributorship statement: AL and SvW set up the study at their respective study sites, coordinated recruitment and data collection, and drafted the manuscript. LC and RW contributed to participant recruitment and data collection. RPCK and RM provided expertise on neuropsychological assessments. ZM, NPR and MN provided expertise on biofluid laboratory analysis, and supervised the processing of blood samples. MCM, DJT, AMT, and AvdK contributed their expertise in neuroimaging. ZM, NPR, HSM, and F-EdL are the principal investigators, secured funding and designed the overall study. All authors reviewed and revised the manuscript for intellectual content and approved the final manuscript.

Competing interests: The authors declare no conflicts of interest.

Funding: This work was supported by a joint grant from the British Heart Foundation and Dutch Heart Foundation (ref: SP/F/22/150028), and jointly sponsored by the Cambridge University Hospitals Foundation NHS Trust and the University of Cambridge in the UK, and the Radboud University Medical Centre in the Netherlands. Infrastructural support was provided by Cambridge British Heart Foundation Centre of Research Excellence (RE/18/1/34212) and Cambridge University Hospitals NIHR Biomedical Research Centre (BRC-1215-20014).

Acknowledgements: The University of Minnesota Center for Magnetic Resonance Research provided us with the pulse sequence software for DTI, which is gratefully acknowledged. We thank our research volunteers for their participation.

For peer review only

REFERENCES

1 Wardlaw JM, Smith C, Dichgans M. Mechanisms underlying sporadic cerebral small vessel disease: insights from neuroimaging. *Lancet Neurology*. 2013;12:483–97.

2 Sachdev P, Kalaria R, O'Brien J, *et al.* Diagnostic criteria for vascular cognitive disorders: A VASCOG statement. *Alzheimer Dis Assoc Disord*. 2014;28:206–18.

3 Gorelick PB, Scuteri A, Black SE, *et al.* Vascular contributions to cognitive impairment and dementia: A statement for healthcare professionals from the American Heart Association/American Stroke Association. *Stroke*. 2011;42:2672–713.

4 Markus HS, Erik de Leeuw F. Cerebral small vessel disease: Recent advances and future directions. *International Journal of Stroke*. 2023;18:4–14.

5 Williamson JD, Pajewski NM, Auchus AP, *et al.* Effect of Intensive vs Standard Blood Pressure Control on Probable Dementia: A Randomized Clinical Trial. *JAMA*. 2019;321:553–61.

6 Wardlaw JM, Smith C, Dichgans M. Small vessel disease: mechanisms and clinical implications. *Lancet Neurol*. Published Online First: 2019. doi: 10.1016/S1474-4422(19)30079-1

7 Low A, Mak E, Rowe JB, *et al.* Inflammation and cerebral small vessel disease: A systematic review. *Ageing Res Rev*. 2019;53:100916.

8 Low A, Mak E, Malpetti M, *et al.* In vivo neuroinflammation and cerebral small vessel disease in mild cognitive impairment and Alzheimer's disease. *J Neurol Neurosurg Psychiatry*. 2021;92:45–52.

9 Walsh J, Tozer DJ, Sari H, *et al.* Microglial activation and blood–brain barrier permeability in cerebral small vessel disease. *Brain*. 2021;144:1361–71.

10 Simpson JE, Ince PG, Higham CE, *et al.* Microglial activation in white matter lesions and nonlesional white matter of ageing brains. *Neuropathol Appl Neurobiol*. 2007;33:670–83.

11 Noz MP, Ter Telgte A, Wiegertjes K, *et al.* Trained immunity characteristics are associated with progressive cerebral small vessel disease. *Stroke*. 2018;49:2910–7.

12 Noz MP, Ter Telgte A, Wiegertjes K, *et al.* Pro-inflammatory Monocyte Phenotype During Acute Progression of Cerebral Small Vessel Disease. *Front Cardiovasc Med*. 2021;8:639361.

13 Riksen NP, Bekkering S, Mulder WJM, *et al.* Trained immunity in atherosclerotic cardiovascular disease. *Nature Reviews Cardiology 2023*. 2023;1–13.

14 Wendeln A-C, Degenhardt K, Kaurani L, *et al.* Innate immune memory in the brain shapes neurological disease hallmarks. *Nature*. 2018;556:332–8.

For peer review only - http://bmjopen.bmj.com/site/about/guidelines.xhtml

15 Tomimoto H, Akiguchi I, Suenaga T, *et al.* Alterations of the blood-brain barrier and glial cells in white-matter lesions in cerebrovascular and Alzheimer's disease patients. *Stroke*. 1996;27:2069–74.

16 Wardlaw JM, Doubal F, Armitage P, *et al.* Lacunar stroke is associated with diffuse blood-brain barrier dysfunction. *Ann Neurol*. 2009;65:194–202.

17 Taheri S, Gasparovic C, Huisa BN, *et al.* Blood-Brain Barrier Permeability Abnormalities in Vascular Cognitive Impairment. *Stroke*. 2011;42:2158.

18 Traylor M, Persyn E, Tomppo L, *et al.* Genetic basis of lacunar stroke: a pooled analysis of individual patient data and genome-wide association studies. *Lancet Neurol*. 2021;20:351–61.

19 Topakian R, Barrick TR, Howe FA, *et al.* Blood–brain barrier permeability is increased in normal-appearing white matter in patients with lacunar stroke and leucoaraiosis. *J Neurol Neurosurg Psychiatry*. 2010;81:192–7.

20 Jalal FY, Yang Y, Thompson JF, *et al.* Hypoxia-induced neuroinflammatory white-matter injury reduced by minocycline in SHR/SP. *Journal of Cerebral Blood Flow and Metabolism*. 2015;35:1145–53.

21 Van Norden AGW, De Laat KF, Van Dijk EJ, *et al.* Diffusion tensor imaging and cognition in cerebral small vessel disease The RUN DMC study☆. *BBA - Molecular Basis of Disease*. 2012;1822:401–7.

22 Wardlaw JM, Smith EE, Biessels GJ, *et al.* Neuroimaging standards for research into small vessel disease and its contribution to ageing and neurodegeneration. *Lancet Neurol*. 2013;12:822–38.

23 Duering M, Biessels GJ, Brodtmann A, *et al.* Neuroimaging standards for research into small vessel disease—advances since 2013. *Lancet Neurol*. 2023;0. doi: 10.1016/S1474-4422(23)00131-X

24 van Norden AGW, de Laat KF, Gons RAR, *et al.* Causes and consequences of cerebral small vessel disease. The RUN DMC study: A prospective cohort study. Study rationale and protocol. *BMC Neurol*. 2011;11:1–8.

25 Staszewski J, Piusińska-Macoch R, Brodacki B, *et al.* IL-6, PF-4, sCD40 L, and homocysteine are associated with the radiological progression of cerebral small-vessel disease: A 2-year follow-up study. *Clin Interv Aging*. 2018;13:1135–41.

26 Bonita R, Beaglehole R. Modification of Rankin Scale: Recovery of motor function after stroke. *Stroke*. 1988;19:1497–500.

For peer review only

For peer review only - http://bmjopen.bmj.com/site/about/guidelines.xhtml

27 Moeller S, Yacoub E, Olman CA, *et al.* Multiband multislice GE-EPI at 7 tesla, with 16-fold acceleration using partial parallel imaging with application to high spatial and temporal whole-brain fMRI. *Magn Reson Med*. 2010;63:1144–53.

28 Xu J, Moeller S, Auerbach EJ, *et al.* Evaluation of slice accelerations using multiband echo planar imaging at 3T. *Neuroimage*. 2013;83:991–1001.

29 Feinberg DA, Moeller S, Smith SM, *et al.* Multiplexed Echo Planar Imaging for Sub-Second Whole Brain FMRI and Fast Diffusion Imaging. *PLoS One*. 2010;5:e15710-.

30 Smith SM, Jenkinson M, Woolrich MW, *et al.* Advances in functional and structural MR image analysis and implementation as FSL. *Neuroimage*. 2004;23:S208–19.

31 Baykara E, Gesierich B, Adam R, *et al.* A Novel Imaging Marker for Small Vessel Disease Based on Skeletonization of White Matter Tracts and Diffusion Histograms. *Ann Neurol*. 2016;80:581–92.

32 Patlak CS, Blasberg RG. Graphical evaluation of blood-to-brain transfer constants from multiple-time uptake data. Generalizations. *Journal of Cerebral Blood Flow and Metabolism*. 1985;5:584–90.

33 Janssen E, ter Telgte A, Verburgt E, *et al.* The Hyperintense study: Assessing the effects of induced blood pressure increase and decrease on MRI markers of cerebral small vessel disease: Study rationale and protocol. *Eur Stroke J*. 2022;7:331–8.

34 Fazekas F. Magnetic Resonance Signal Abnormalities in Asymptomatic Individuals: Their Incidence and Functional Correlates. *Eur Neurol*. 1989;29:164–8.

35 Potter GM, Chappell FM, Morris Z, *et al.* Cerebral perivascular spaces visible on magnetic resonance imaging: development of a qualitative rating scale and its observer reliability. *Cerebrovasc Dis*. 2015;39:224–31.

36 Lippi G, Daves M, Mattiuzzi C. Interference of medical contrast media on laboratory testing. *Biochem Med (Zagreb)*. 2014;24:80–8.

37 Scheiermann C, Kunisaki Y, Frenette PS. Circadian control of the immune system. *Nature Reviews Immunology 2013 13:3*. 2013;13:190–8.

38 Lezak MD, Howieson DB, Bigler ED, *et al.* *Neuropsychological assessment*. 5th ed. New York, NY, US: Oxford University Press 2012.

39 Yesavage JA, Brink TL, Rose TL, *et al.* Development and validation of a geriatric depression screening scale: A preliminary report. *J Psychiatr Res*. 1982;17:37–49.

40 Marin RS, Biedrzycki RC, Firinciogullari S. Reliability and validity of the apathy evaluation scale. *Psychiatry Res*. 1991;38:143–62.

For peer review only - http://bmjopen.bmj.com/site/about/guidelines.xhtml

41 Krupp LB, Larocca NG, Muir Nash J, *et al.* The Fatigue Severity Scale: Application to Patients With Multiple Sclerosis and Systemic Lupus Erythematosus. *Arch Neurol*. 1989;46:1121–3.

42 Ganguli M, Sun Z, Mcdade E, *et al.* That's Inappropriate! Social Norms in an Older Population-based Cohort. *Alzheimer Dis Assoc Disord*. 2018;32:150–5.

43 Zeestraten EA, Benjamin P, Lambert C, *et al.* Application of Diffusion Tensor Imaging Parameters to Detect Change in Longitudinal Studies in Cerebral Small Vessel Disease. *PLoS One*. 2016;11:e0147836.

For peer review only

For peer review only - http://bmjopen.bmj.com/site/about/guidelines.xhtml

LIST OF FIGURES

Figure 1. Overview of study components.

Figure 2. Study flowchart; based on planned sample sizes.

For peer review only

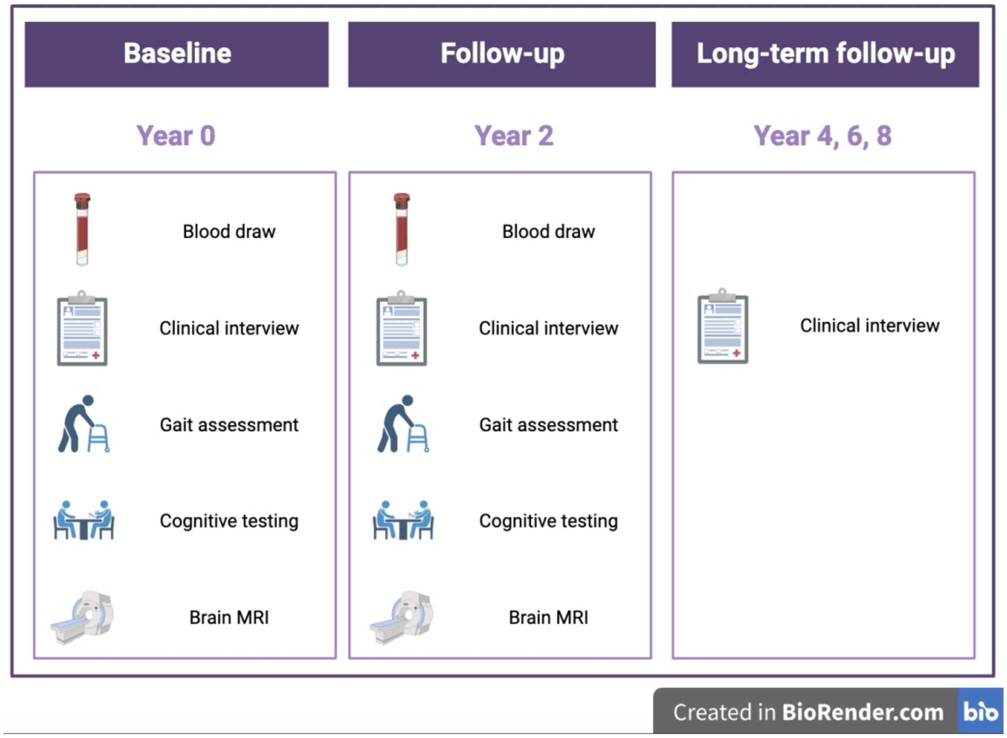

Figure 1. Overview of study components.

262x194mm (144 x 144 DPI)

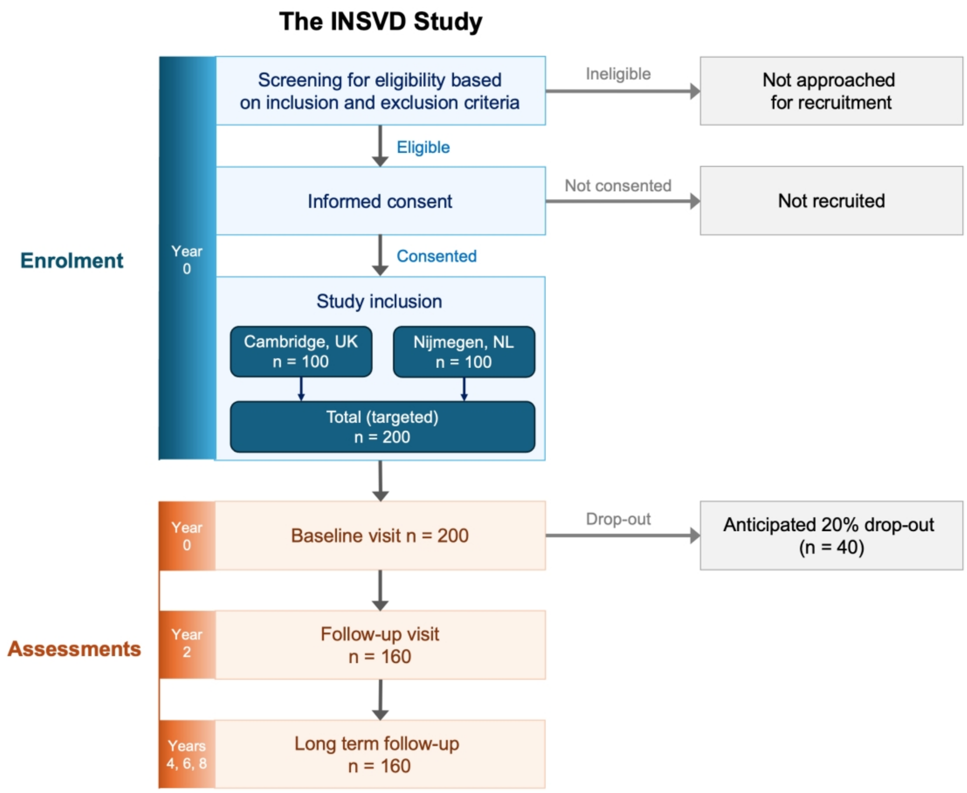

Figure 2. Study flowchart; based on planned sample sizes.

338x280mm (150 x 150 DPI)

Supplementary Table 1. MRI acquisition parameters.

	T1		FLAIR		T2* gradient echo		DTI		IVIM		DCE	
					SWI	QSM						
**Study site**	C	N	C	N	C	N	C	N	C	N	C	N
**Sequence**	3D MPRAGE		3D SPACE		3D SPGR		Single-shot spin-echo EPI		NA	Single-shot spin-echo EPI	3D SPGR	
**TR,** ms	2300		6000	5000	29	40	2433		NA	3386	15	
**TE,** ms	2.98		383	394	20	3.5, 8.6, 13.7, 18.9, 24.0, 29.2, 34.3	75.6		NA	60.6	1.75	
**TI,** ms	900		2100	1800	NA	NA	NA		NA	NA	NA	
**Flip angle**	9°		multiple	multiple	15°	15°	80°		NA	90°	5°, 10°, 15°, 21°, 26°	
**Fat suppression**	None		None	None	None	WEX	FatSat		NA	FatSat	None	
**FOV** mm^2	256×248		256×256	250×250	256×256	256×256	192×192		NA	212×212	240×240	
**Voxel size** mm^3	1×1×1		1×1×1	0.5×0.5×1	0.5×0.5×2	1×1×1	1.7×1.7×1.8		NA	2×2×2	1.9×1.9×4.0	
**Slices**	176		192	176	72	176	76		NA	74	16	
**Slice orientation**	SAG		SAG	SAG	TRA	SAG	TRA		NA	TRA	TRA	
**Diffusion b-values** s/mm^2	NA		NA	NA	NA	NA	0, 300, 1000, 2000		NA	0, 5, 10, 15, 20, 30, 40, 50, 60, 100, 200, 400, 600, 800, 1000	NA	
**Diffusion gradient directions**	NA		NA	NA	NA	NA	6, 8, 30, 60		NA	6	NA	
**Parallel imaging accel. Factor**	2	3	2	2	2	6	NA		NA	2	NA	
**Multiband accel. Factor**	NA		NA	NA	NA	NA	4		NA	2	NA	
**Acquisition time,** mm:ss	05:12	03:01	05:44	04:42	04:35	05:06	04:28		NA	05:39	21:12	
**Temporal res.** mm:ss	NA		NA	NA	NA	NA	NA		NA	NA	02:39	

Abbreviations: C = Cambridge, DCE = dynamic contrast-enhanced MRI, DTI = diffusion tensor imaging (multi-shell with 98 gradient directions and 6 b=0 values), *FLAIR = fluid-attenuated inversion recovery, FOV = field of view, GRAPPA = GeneRalized Autocalibrating Partial Parallel Acquisition, IVIM = Intravoxel Incoherent Motion, N = Nijmegen, SAG = sagittal, SWI = susceptibility-weighted imaging, TI = inversion time, TE = echo time, TR = repetition time, TRA = transversal*.

Supplementary Table 2. Neuropsychological assessment measures.

Measured construct	Instrument	Mode of administration	Type of measure
Premorbid intelligence	National/Dutch Adult Reading Test	Verbal	# correctly pronounced
Processing speed	Trail Making Test A	Written	time, # errors
	Symbol Digit Modalities Test	Written	# completed in 90s
Working memory	Digit Span Forwards and Backwards	Verbal	# correct trials
	Story Recall – Immediate Recall	Verbal	# correct elements recalled
Executive function	Trail Making Test B	Verbal	time, # errors
	Brixton Spatial Anticipation Test	Verbal	# errors
Visuospatial ability	Rey-Osterrieth Complex Figure Copy	Written	# correct elements drawn
Memory	Story Recall – Delayed Recall	Verbal	# correct elements recalled
	Rey-Osterrieth Complex Figure Recall	Written	# correct elements recalled
Depression	Geriatric Depression Scale (30-item version)	Self-administered questionnaire	Yes/No
Apathy	Apathy Evaluation Scale – Self	Self-administered questionnaire	4-point scale
	Apathy Evaluation Scale – Informant	Informant-rated questionnaire	4-point scale
Fatigue	Fatigue Severity Scale	Self-administered questionnaire	7-point scale
Social cognition	Social Norms Questionnaire	Self-administered questionnaire	Yes/No

For peer review only

Reporting checklist for cohort study.

Based on the STROBE cohort guidelines.

Instructions to authors

Complete this checklist by entering the page numbers from your manuscript where readers will find each of the items listed below.

Your article may not currently address all the items on the checklist. Please modify your text to include the missing information. If you are certain that an item does not apply, please write "n/a" and provide a short explanation.

Upload your completed checklist as an extra file when you submit to a journal.

In your methods section, say that you used the STROBE cohortreporting guidelines, and cite them as:

von Elm E, Altman DG, Egger M, Pocock SJ, Gotzsche PC, Vandenbroucke JP. The Strengthening the Reporting of Observational Studies in Epidemiology (STROBE) Statement: guidelines for reporting observational studies.

		Reporting Item	Page Number
**Title and abstract**			
Title	#1a	Indicate the study's design with a commonly used term in the title or the abstract	1
Abstract	#1b	Provide in the abstract an informative and balanced summary of what was done and what was found	2
**Introduction**			
Background / rationale	#2	Explain the scientific background and rationale for the investigation being reported	4
Objectives	#3	State specific objectives, including any prespecified hypotheses	5
**Methods**			

For peer review only

Study design	#4	Present key elements of study design early in the paper	6
Setting	#5	Describe the setting, locations, and relevant dates, including periods of recruitment, exposure, follow-up, and data collection	6
Eligibility criteria	#6a	Give the eligibility criteria, and the sources and methods of selection of participants. Describe methods of follow-up.	6
Eligibility criteria	#6b	For matched studies, give matching criteria and number of exposed and unexposed	na
Variables	#7	Clearly define all outcomes, exposures, predictors, potential confounders, and effect modifiers. Give diagnostic criteria, if applicable	8-11
Data sources / measurement	#8	For each variable of interest give sources of data and details of methods of assessment (measurement). Describe comparability of assessment methods if there is more than one group. Give information separately for for exposed and unexposed groups if applicable.	8-11
Bias	#9	Describe any efforts to address potential sources of bias	7,9
Study size	#10	Explain how the study size was arrived at	7
Quantitative variables	#11	Explain how quantitative variables were handled in the analyses. If applicable, describe which groupings were chosen, and why	12
Statistical methods	#12a	Describe all statistical methods, including those used to control for confounding	12
Statistical methods	#12b	Describe any methods used to examine subgroups and interactions	10
Statistical methods	#12c	Explain how missing data were addressed	7
Statistical methods	#12d	If applicable, explain how loss to follow-up was addressed	7
Statistical methods	#12e	Describe any sensitivity analyses	12

For peer review only

Results

Participants	#13a	Report numbers of individuals at each stage of study—eg numbers potentially eligible, examined for eligibility, confirmed eligible, included in the study, completing follow-up, and analysed. Give information separately for for exposed and unexposed groups if applicable.	7
Participants	#13b	Give reasons for non-participation at each stage	na
Participants	#13c	Consider use of a flow diagram	na
Descriptive data	#14a	Give characteristics of study participants (eg demographic, clinical, social) and information on exposures and potential confounders. Give information separately for exposed and unexposed groups if applicable.	na
Descriptive data	#14b	Indicate number of participants with missing data for each variable of interest	na
Descriptive data	#14c	Summarise follow-up time (eg, average and total amount)	6,7
Outcome data	#15	Report numbers of outcome events or summary measures over time. Give information separately for exposed and unexposed groups if applicable.	na
Main results	#16a	Give unadjusted estimates and, if applicable, confounder-adjusted estimates and their precision (eg, 95% confidence interval). Make clear which confounders were adjusted for and why they were included	na
Main results	#16b	Report category boundaries when continuous variables were categorized	9
Main results	#16c	If relevant, consider translating estimates of relative risk into absolute risk for a meaningful time period	na

For peer review only

Other analyses	#17	Report other analyses done—eg analyses of subgroups and interactions, and sensitivity analyses	12
**Discussion**			
Key results	#18	Summarise key results with reference to study objectives	na
Limitations	#19	Discuss limitations of the study, taking into account sources of potential bias or imprecision. Discuss both direction and magnitude of any potential bias.	3
Interpretation	#20	Give a cautious overall interpretation considering objectives, limitations, multiplicity of analyses, results from similar studies, and other relevant evidence.	na
Generalisability	#21	Discuss the generalisability (external validity) of the study results	na
**Other Information**			
Funding	#22	Give the source of funding and the role of the funders for the present study and, if applicable, for the original study on which the present article is based	2,13

None The STROBE checklist is distributed under the terms of the Creative Commons Attribution License CC-BY. This checklist can be completed online using https://www.goodreports.org/, a tool made by the EQUATOR Network in collaboration with Penelope.ai

For peer review only
