# Supplementary material for: Immune regulation and blood–brain barrier permeability in cerebral small vessel disease: study protocol of the INflammation and Small Vessel Disease (INSVD) study – a multicentre prospective cohort study
Source: BMJ Open. 2024 Feb 26;14(2):e084303. doi: 10.1136/bmjopen-2024-084303 (PMC10900331; doi:10.1136/bmjopen-2024-084303)
Supplement: Reviewer comments [file bmjopen-2024-084303.reviewer_comments.pdf]

PEER REVIEW HISTORY

BMJ Open publishes all reviews undertaken for accepted manuscripts. Reviewers are asked to complete a checklist review form (**http://bmjopen.bmj.com/site/about/resources/checklist.pdf**) and are provided with free text boxes to elaborate on their assessment. These free text comments are reproduced below.

ARTICLE DETAILS

**TITLE (PROVISIONAL)**	Immune regulation and blood-brain barrier permeability in cerebral small vessel disease – study protocol of the INflammation and Small Vessel Disease (INSVD) study, a multicentre prospective cohort study
**AUTHORS**	Low, Audrey; van Winden, Sanne; Cai, Lupei; Kessels, Roy; Maas, Marnix C.; Morris, Robin; Nus, Meritxell; Tozer, Daniel; Tuladhar, Anil; van der Kolk, Anja; Wolters, Rowan; Mallat, Ziad; Riksen, Niels; Markus, Hugh; de Leeuw, Frank-Erik

This article was not externally reviewed at BMJ Open. Protocols that have been independently assessed prior to submission to BMJ Open are usually fast-tracked to publication on the grounds that further substantial changes will not be possible. This independent assessment will usually be external, independent review for both a substantial grant award from a non-commercial or government funder and ethics approval.
